# Supplementary material for: Ferroptosis spreads to neighboring cells via plasma membrane contacts
Source: Nat Commun. 2025 Mar 26;16:2951. doi: 10.1038/s41467-025-58175-w (PMC11947162; doi:10.1038/s41467-025-58175-w)
Supplement: Supplementary file 1 — Supplementary Information [file 41467_2025_58175_MOESM1_ESM.pdf]

Supplementary Figures

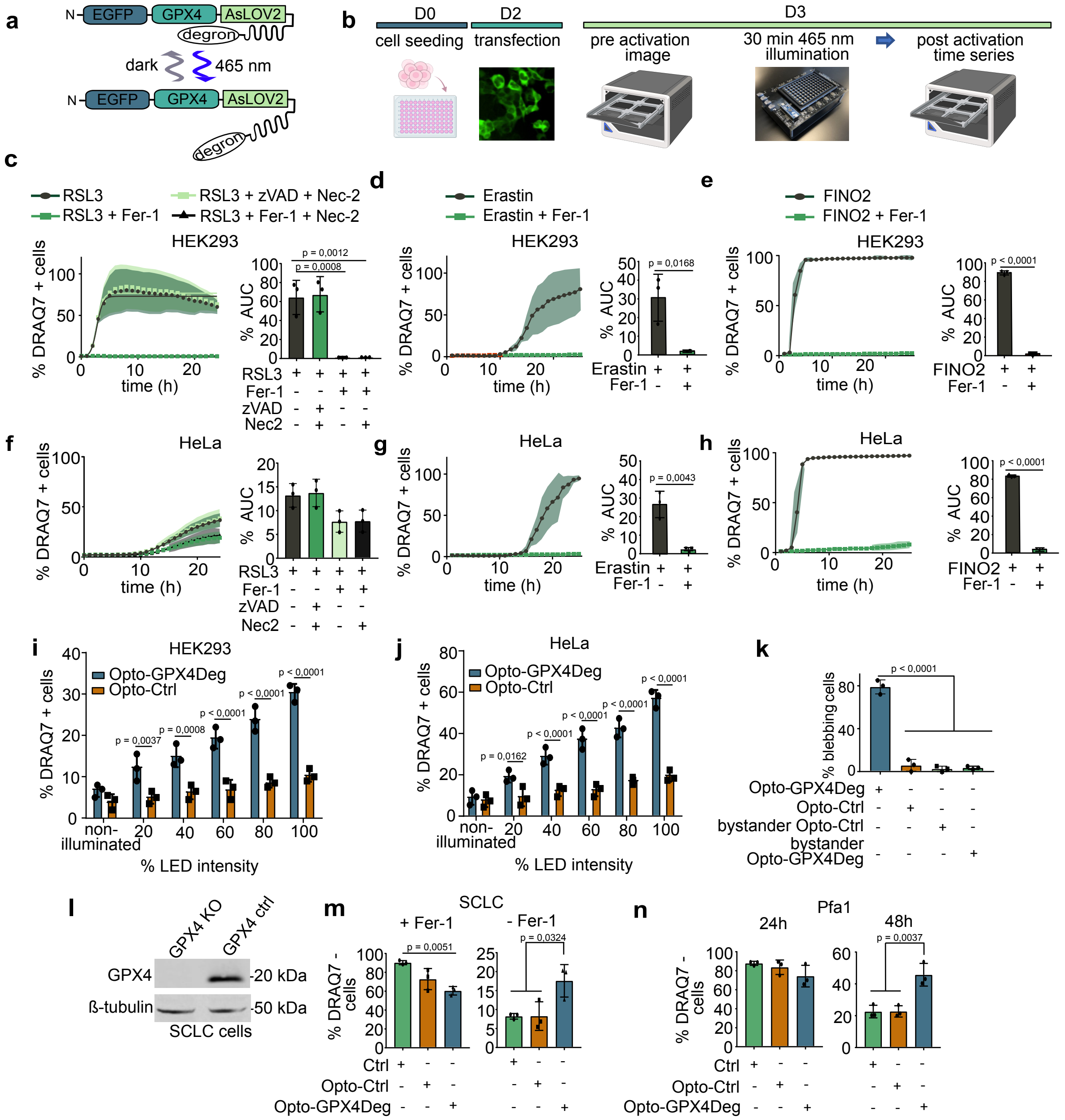

Supplementary Figure 1: Characterization of the Opto-GPX4Deg construct. (a) Scheme of Opto-GPX4Deg. The construct encodes for a fusion protein with GPX4 tagged with EGFP at the N-terminus and a LOV domain with a degron sequence at the C-terminus, which only becomes accessible upon blue light illumination. **(b)** Workflow of high-throughput optogenetic experiments: On day 0, 5000 HeLa or HEK293 cells/well were seeded into 96-well plates. On day 2, the cells were transfected either with Opto-Ctrl or Opto-GPX4Deg. On day 3, a pre-activation image was acquired before illuminating the bulks for 30 min with 100% 465 nm LED intensity using an optoPlate-96. Subsequently, a post activation time series was acquired using a S3-Incucyte. Created in BioRender. Garcia, A. (2025) https://BioRender.com/l81y945. **(c-h)** Cell death kinetics of HeLa or HEK293 cells treated as indicated (4 µM RSL3), (4 µM RSL3 + 4 µM Fer-1), (4 µM RSL3 + 4 µM Fer-1 + 10µM zVAD), (4 µM RSL3 + 10

µM Nec-2 + 10µM zVAD), (20 µM Erastin), (20 µM Erastin + 20 µM Fer-1), (30 µM FINO2) or (30 µM FINO2 + 30 µM Fer-1) and assessed via DRAQ7 staining using an IncuCyte S3. %AUC of different treatments. Statistical analysis by two-sided parametric t-test or two-sided one-way ANOVA corrected for multiple comparisons using Tukey's multiple comparison test. Exact p-values are shown. All experiments were performed with three independent biological replicates (n=3). Values as mean ± SD. **(i, j)** Cell death is proportional to illumination intensity in cells expressing Opto-GPX4Deg, but not in Opto-Ctrl. Experiments as in **Fig. 1a,b** except that a gradient of 465 nm LED intensities was used. Statistical analysis by two-sided multiple t-test using the Holm-Sidak method. Statistical analysis by two-sided one-way ANOVA corrected for multiple comparisons using Tukey's multiple comparison test. Exact p-values are shown. All experiments were performed with three independent biological replicates (n=3). Values as mean ± SD. **(k)** Quantification of the % blebbing cells from **Fig. 2b** at 25 min after illumination. Bystander refers to non-expressing bystander cells exposed to activating illumination within populations transfected with Opto-GPX4Deg or Opto-Ctrl. Statistical analysis by two-sided one-way ANOVA corrected for multiple comparisons using Tukey's multiple comparison test. Exact p-values are shown. All experiments were performed with three independent biological replicates (n=3). Values as mean ± SD. **(l-n)** Opto-GPX4Deg retains partial GPX4 activity. **(l)** Representative WB showing that the SCLC GPX4 KO cells are GPX4 deficient. **(m)** % DRAQ7 negative GPX4 KO SCLC cells transfected or not with Opto-GPX4Deg or Opto-Ctrl and treated or not with 5 µM Fer-1. Statistical analysis by two-sided one-way ANOVA corrected for multiple comparisons using Tukey's multiple comparison test. Exact p-values are shown. All experiments were performed with three independent biological replicates (n=3). Values as mean ± SD. **(n)** Tamoxifen inducible Pfa1 GPX4 KO cells transfected with Opto-GPX4Deg show increased viability compared to non-transfected cells after 48h 1 µM tamoxifen treatment. Statistical analysis by two-sided one-way ANOVA corrected for multiple comparisons using Tukey's multiple comparison test. Exact p-values are shown. All experiments were performed with three independent biological replicates (n=3). Values are displayed as mean ± SD.

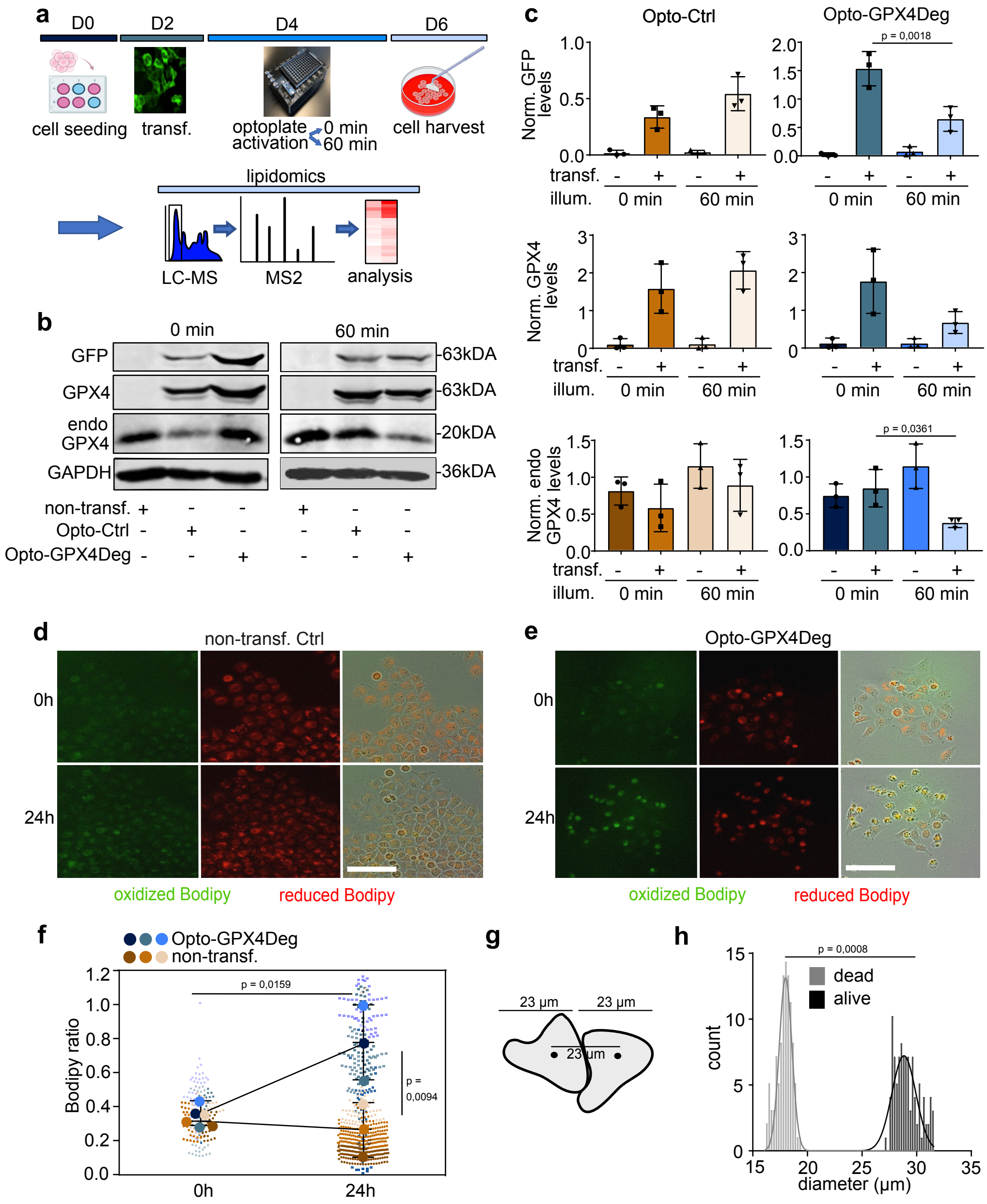

Supplementary Figure 2: Opto-GPX4Deg activation induces ferroptosis. (a) Workflow of lipidomic experiments: On day 0, 1x10^5 HeLa cells/well were seeded into 6-well plates. On day 2, HeLa cells were transfected either with Opto-Ctrl, Opto-GPX4Deg or not transfected. Bulks were illuminated or not for 60 min with 100% 465 nm LED intensity using an optoPlate-96. After 48h, the cells were harvested and samples were prepared for WB and lipidomics. Created in BioRender. Garcia, A. (2025) https://BioRender.com/p30n108. **(b)** Representative WB showing the illumination-dependent degradation of the Opto-GPX4Deg construct. GAPDH used as loading control. **(c)** Quantification of the GFP, GPX4 and endogenous GPX4 proteins levels of the WB in **(b).** Protein levels normalized to loading control. –, non-transfected, or transfected as indicated with Opto-Ctrl or Opto-GPX4Deg. Statistical analysis by two-sided one-way ANOVA corrected for multiple comparisons using Tukey's multiple comparison test (rest) or two-sided parametric t-test (endo GPX4). Exact p-values are

shown. All experiments were performed with three independent biological replicates (n=3). Values as mean ± SD. **(d, e)** Representative images of C11-Bodipy oxidation (green) upon illumination of cells expressing or not Opto-GPX4Deg. Reduced C11-Bodipy shown in red. **(f)** For calculation of the C11-Bodipy ratio, the green (oxidized) C11-Bodipy signal divided by the sum of red and green C11-Bodipy signal (total). The big dots represent replicate averages, the small dots represent single cells in individual replicates. Statistical analysis by two-sided one-way ANOVA corrected for multiple comparisons using Tukey's multiple comparison test. Exact p-values are shown. All experiments were performed with three independent biological replicates (n=3). Values are displayed as mean ± SD. **(g)** Scheme indicating the average diameter of HeLa cells as well as the average distance between adjacent HeLa cells. **(h)** Quantification of the distribution of cell diameters of living and dead HeLa cells from experiments in **Fig.3 a-d.** Statistical analysis by two-sided parametric t-test. Exact p-values are shown. All experiments were performed with three independent biological replicates (n=3). Values are displayed as mean ± SD.

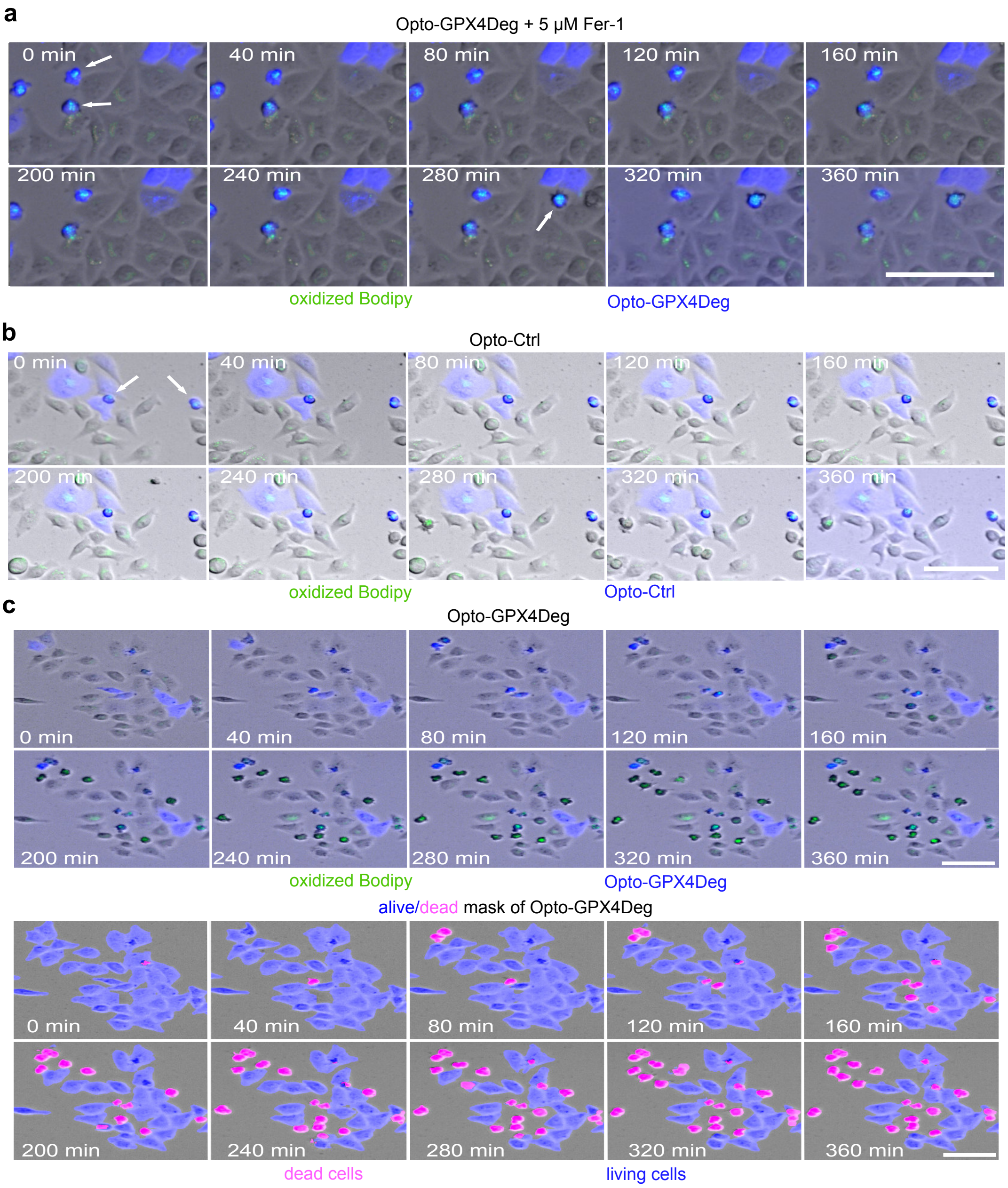

Supplementary Figure 3: Ferrostatin blocks cell death propagation to neighboring cells. (a,b) Images from experiments in **Fig.3 H-N.** Representative time series of C11-Bodipy oxidation (green) and cell death for the indicated conditions. Blue signal indicates Opto-GPX4Deg or Opto-Ctrl expression. White arrows indicate dead, oxidized C11-Bodipy positive cells, whereas black arrows indicate dead, oxidized C11-Bodipy negative cells. All experiments were performed with three independent biological replicates (n=3). Scale bar, 100 µm. **(c)** AI-derived alive/dead mask allows the assessment of cell death in microscopy images. Upper panel, blue signal indicates Opto-GPX4Deg expression, green signal indicates C11-Bodipy oxidation. Lower panel, cell death detection by AI-based software. Living cells are indicated in blue and dead cells in pink. All experiments were performed with three independent biological replicates (n=3). Scale bar, 100 µm.

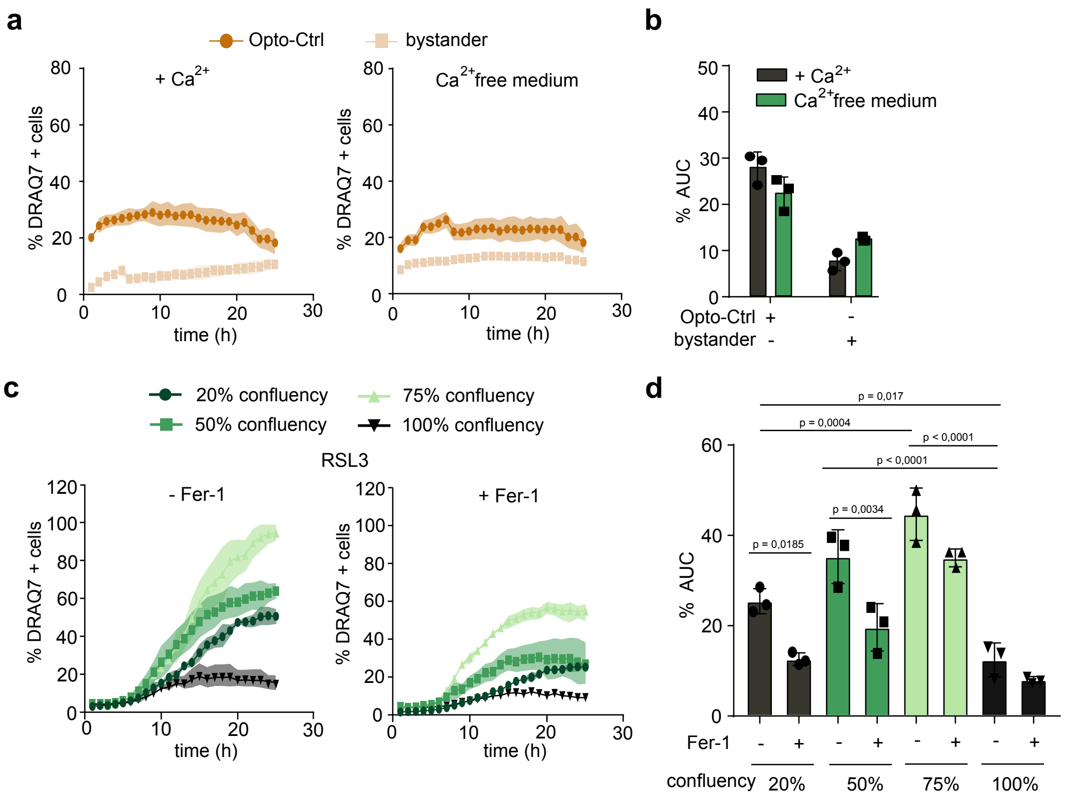

Supplementary Figure 4: Effect of calcium and cell confluency on ferroptosis sensitivity. (a) %DRAQ7 positive cells over time for indicated cell populations with (left) or without Ca^{2+} in the medium (right). **(b)** %AUC for populations in **(a)**. **(c)** %DRAQ7 positive cells over time for HeLa cells seeded at indicated confluency and treated with 4 µM RSL3 and 5 µM Fer-1 as indicated. **(d)** %AUC for the different populations. Statistical analysis by two-sided one-way ANOVA corrected for multiple comparisons using Tukey's multiple comparison test. Exact p-values are shown. All experiments were performed with three independent biological replicates (n=3). Values are displayed as mean ± SD.

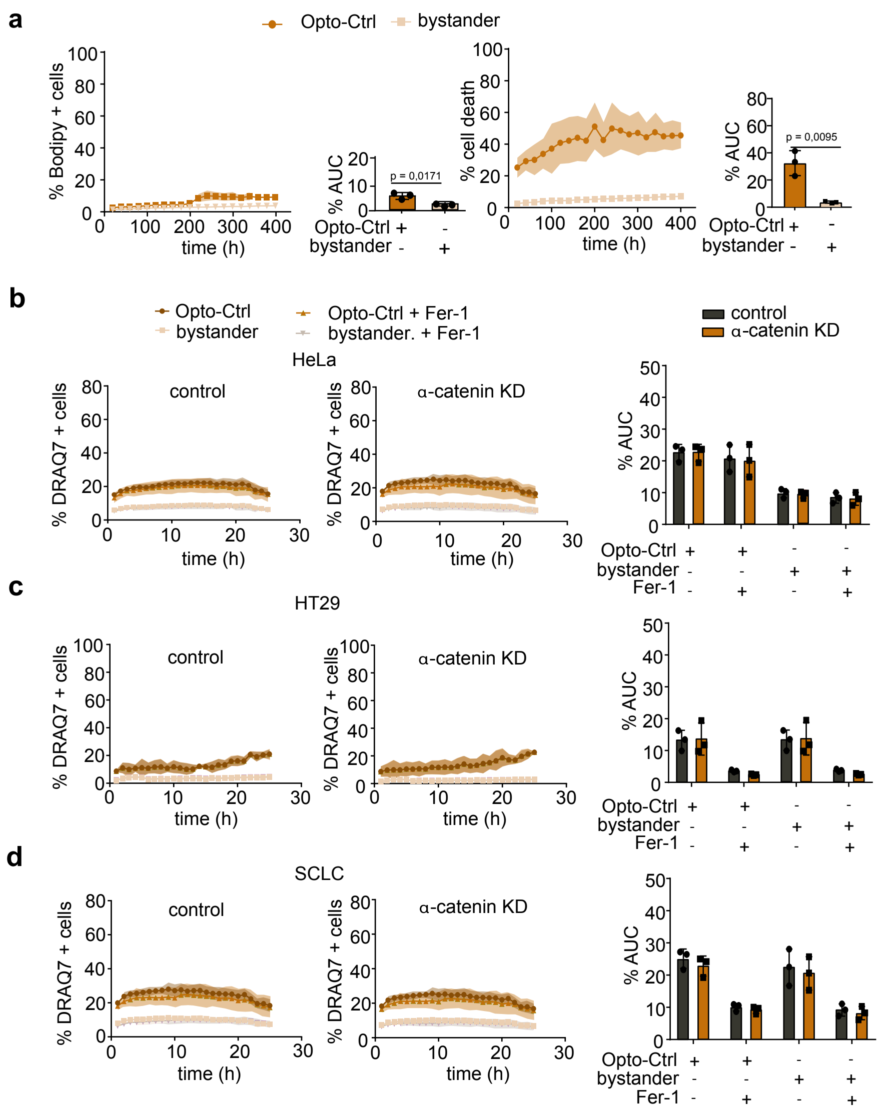

Supplementary Figure 5: Control experiments for α-catenin KD conditions. (a) C11-Bodipy oxidation and cell death kinetics of cells transfected with Opto-Ctrl and treated or not with 5 μM Fer-1. C11-Bodipy positive cells (left) and cell death (right) were quantified using custom made software. %AUC for indicated conditions. All experiments were performed with three independent biological replicates (n=3). Statistical analysis by two-sided parametric t-test. **(b-d)** Cell death kinetics of activated Opto-Ctrl HeLa **(b)**, HT-29 **(c)** or SCLC **(d)** cells transfected with siRNA against α-catenin or scrambled siRNA and treated or not with 5 μM Fer-1. %AUC for the indicated conditions. Statistical analysis by two-sided one-way ANOVA corrected for multiple comparisons using Tukey's multiple comparison test. Exact p-values are shown. All experiments were performed with three independent biological replicates (n=3). Values mean ± SD.

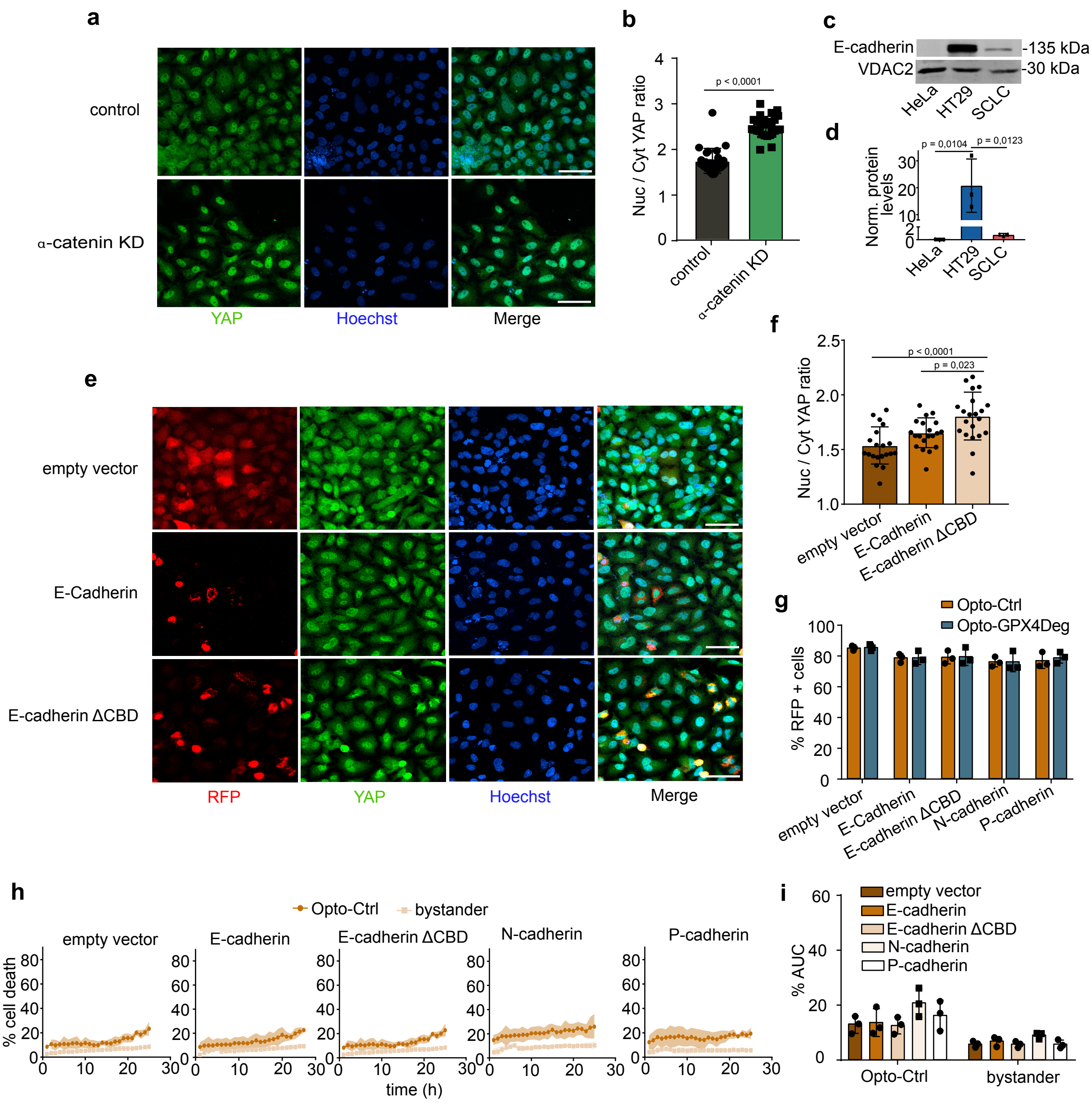

Supplementary Figure 6: Analysis of YAP activation. (a) Immunofluorescence staining of YAP in α-catenin KD or control HeLa cells. Green, YAP; blue, Hoechst as nuclear staining. **(b)** Quantification of nuclear translocation by comparison of nucleus/cytosol (Nuc/Cyt) YAP ratio in α-catenin KD or control HeLa cells. Statistical analysis by two-sided parametric t-test. All experiments were performed with three independent biological replicates (n=3). Values as mean ± SD. **(c)** WB of E-cadherin protein levels in indicated cell lines. **(d)** Quantification of WB in **(c).** Statistical analysis by two-sided one-way ANOVA corrected for multiple comparisons using Tukey's multiple comparison test. All experiments were performed with three independent biological replicates (n=3). Values as mean ± SD. **(e)** Representative immunofluorescence images of YAP translocation to the nucleus. Red, construct expression; green, YAP; blue, Hoechst as nuclear staining. **(f)** Quantification of the nucleus/cytosol (Nuc/Cyt) YAP ratio for the indicated conditions. Statistical analysis by two-sided one-way ANOVA corrected for multiple comparisons using Tukey's multiple

comparison test. All experiments were performed with three independent biological replicates (n=3). Values as mean ± SD. **(g)** Transfection control assessed by quantification of the mRFP expression levels in individual cells using IncuCyte upon optogenetic activation for the experiments in **Fig. 5m-n** and **Fig. S6h-i**. All experiments were performed with three independent biological replicates (n=3). Values as mean ± SD. **(h)** Cell death kinetics of activated Opto-Ctrl and bystander HeLa cells transfected with RFP-tagged empty vector, WT E-cadherin, E-cadherin ΔCBD mutant, WT N-cadherin or WT P-cadherin. **(i)** %AUC for the indicated conditions. Statistical analysis by two-sided one-way ANOVA **(f and i)** or two-sided two-way ANOVA **(g)** corrected for multiple comparisons using Tukey's multiple comparison test. Exact p-values are shown. All experiments were performed with three independent biological replicates (n=3). Values as mean ± SD.

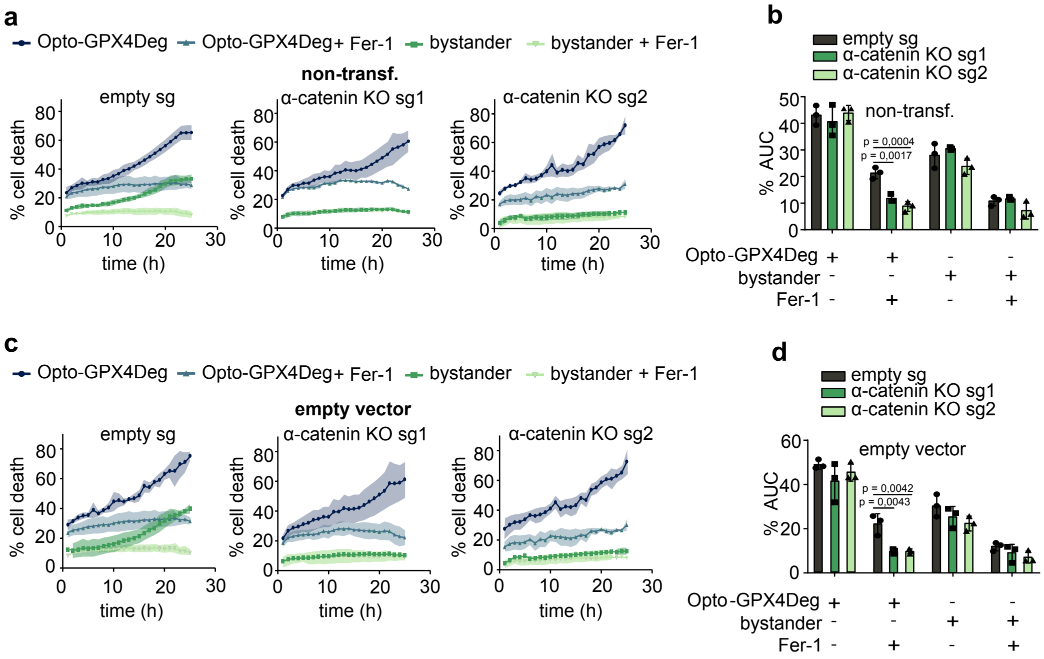

Supplementary Figure 7: Controls for reconstitution experiments in α-catenin KO HeLa cells. (a,c) Kinetics of cell death for activated Opto-GPX4Deg and bystander α-catenin KO HeLa clones (sg1, sg2) or CRISPR control (empty sg) not transfected or transfected with empty vector, treated or not with 5 μM Fer-1. All experiments were performed with three independent biological replicates (n=3). Values as mean ± SD. **(b, d)** %AUC for the indicated cell populations. Statistical analysis by two-sided one-way ANOVA corrected for multiple comparisons using Tukey's multiple comparison test. Exact p-values are shown. All experiments were performed with three independent biological replicates (n=3). Values as mean ± SD.

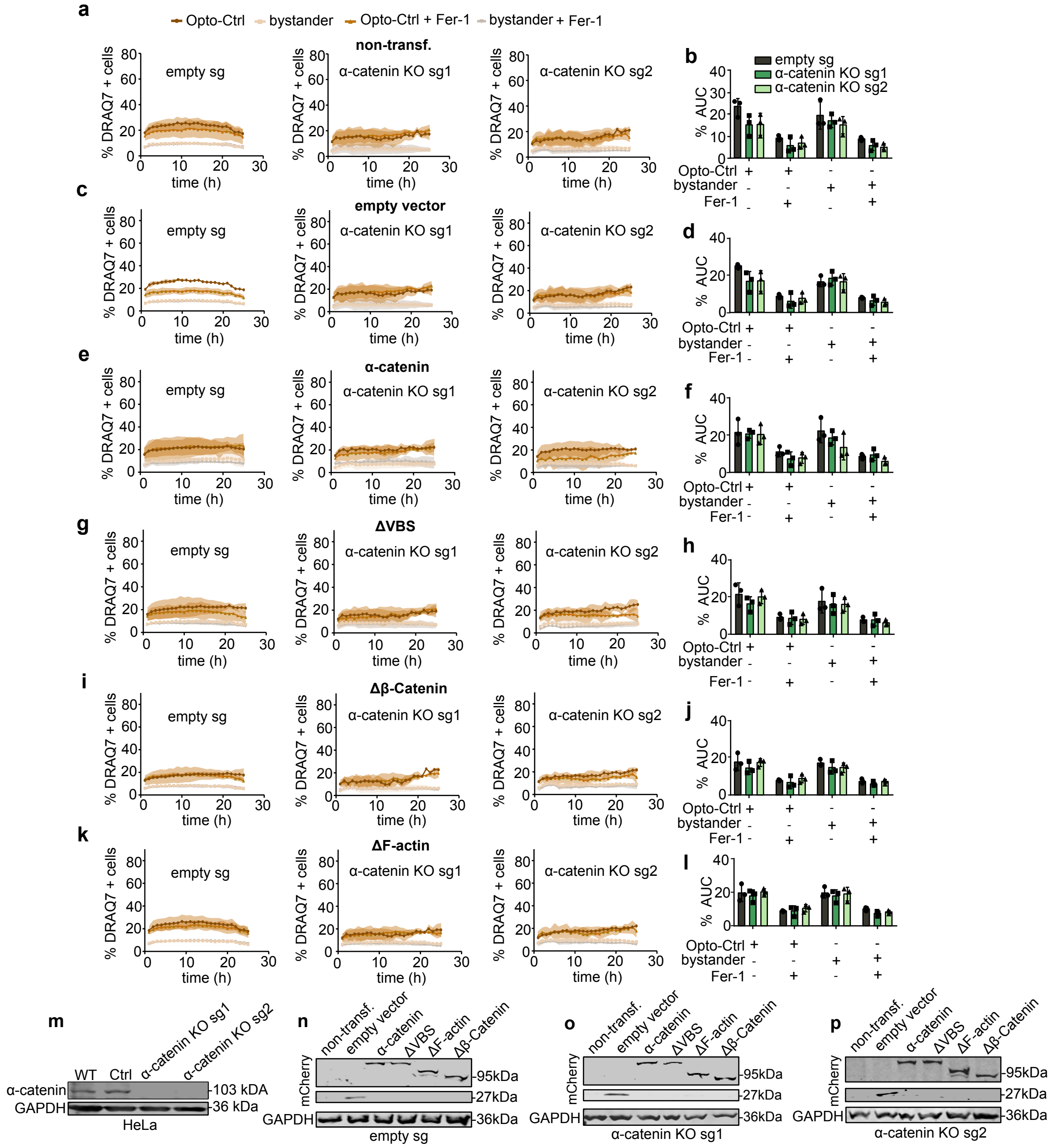

Supplementary Figure 8: Controls for α-catenin reconstitution experiments in α-catenin KO HeLa cells. (a, c, e, g, i, k) Kinetics of cell death for activated Opto-Ctrl and bystander α-catenin KO HeLa clones (sg1, sg2) or CRISPR control (empty sg) not reconstituted or reconstituted with the indicated control, α-catenin WT or indicated α-catenin mutants treated or not with 5 µM Fer-1. All experiments were performed with three independent biological replicates (n=3). Values as mean ± SD. **(b, d, f, h, j, l)** %AUC for the indicated cell populations. Statistical analysis by two-sided one-way ANOVA corrected for multiple comparisons using Tukey's multiple comparison test. Exact p-values are shown. All experiments were performed with three independent biological replicates (n=3). Values as mean ± SD. **(m)** WB of HeLa α-catenin levels in KO cells. **(n-p)** WB of comparable reconstitution levels for the indicated constructs. All experiments were performed with three independent biological replicates (n=3).

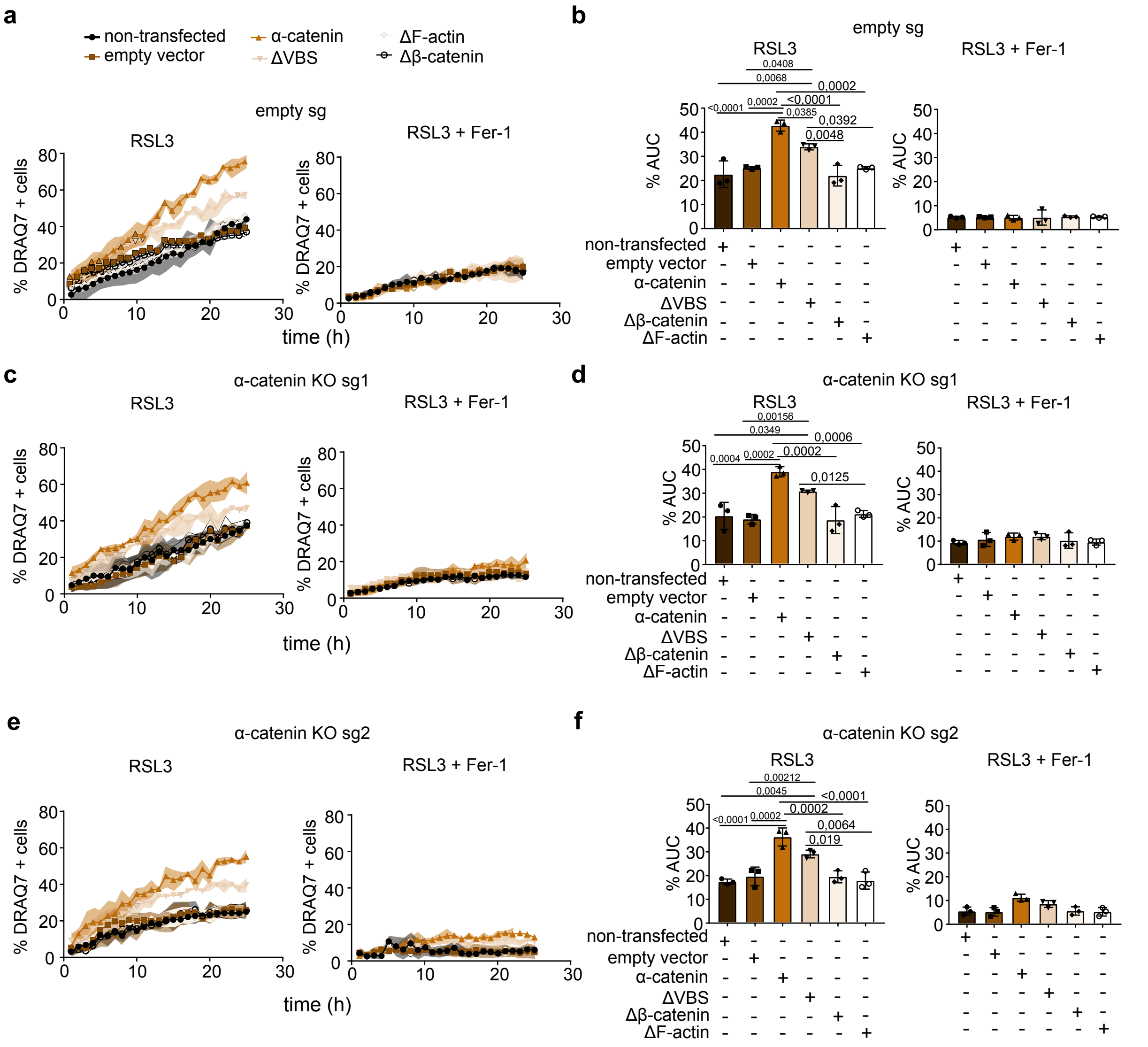

Supplementary Figure 9: Adhesive cell-cell contacts by α-catenin promote ferroptosis sensitivity induced by RSL3. **(a, c, e)** Kinetics of cell death for HeLa KO clones (sg1, sg2) or CRISPR control (empty sg) reconstituted with exogenous WT or mutant α-catenin treated with 6 µM RSL3 or 6 µM RSL3 and 10 µM Fer-1. All experiments were performed with three independent biological replicates (n=3). Values are displayed as mean ± SD. **(b, d, f)** %AUC for indicated cell populations. Statistical analysis by two-sided one-way ANOVA corrected for multiple comparisons using Tukey's multiple comparison test. Exact p-values are shown. All experiments were performed with three independent biological replicates (n=3). Values are displayed as mean ± SD.

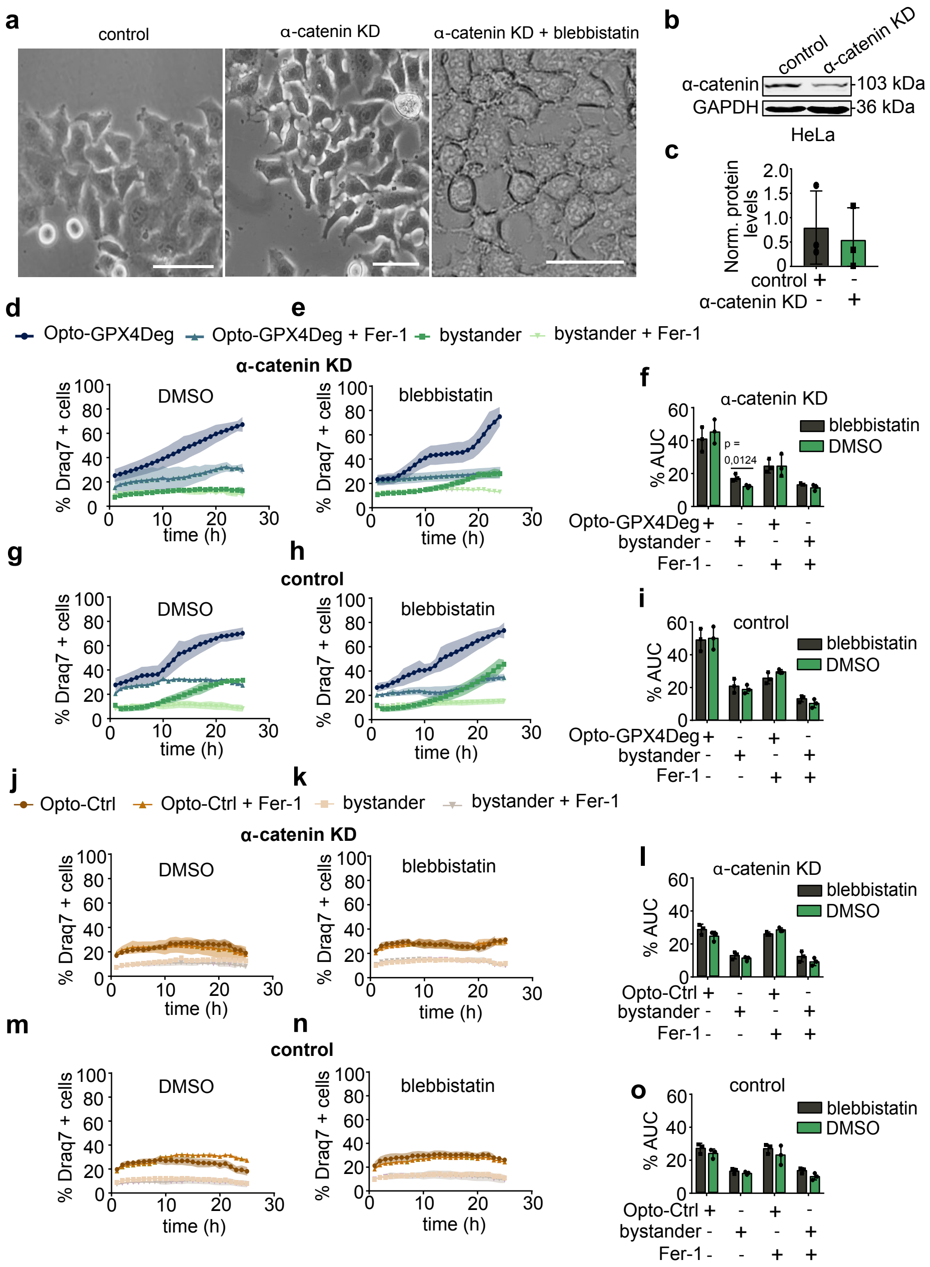

Supplementary Figure 10: Increasing cell-cell contacts promotes ferroptosis propagation. (a) Representative images of HeLa cells transfected with a control siRNA or against α-catenin treated or not with 10 μM blebbistatin for one hour. Scale bar, 30 μm. **(b)** WB analysis of α-catenin KD in entire figure. **(c)** Quantification of WB in **(b)**, normalized to loading control. Statistical analysis by two-sided parametric t-test. All experiments were performed with three independent biological replicates (n=3). Values are displayed as mean ± SD. **(d,e,g,h,j,k,m,n)** Kinetics of cell death for activated Opto-GPX4Deg **(d,e,g,h)** or Opto-Ctrl **(j,k,m,n)** and bystander HeLa cells transfected with siRNA against α-catenin or scrambled siRNA (control), and treated or not with 5 μM Fer-1 and/or 10 μM blebbistatin. All experiments were performed with three independent biological replicates (n=3). Values are displayed as mean ± SD.

(f,l,i,o) %AUC for the indicated populations in **(d,e,g,h,j,k,m,n)**. Statistical analysis by two-sided one-way ANOVA corrected for multiple comparisons using Tukey's multiple comparison test (rest) or two-sided parametric t-test **(f)**. Exact p-values are shown. All experiments were performed with three independent biological replicates (n=3). Values as mean ± SD.

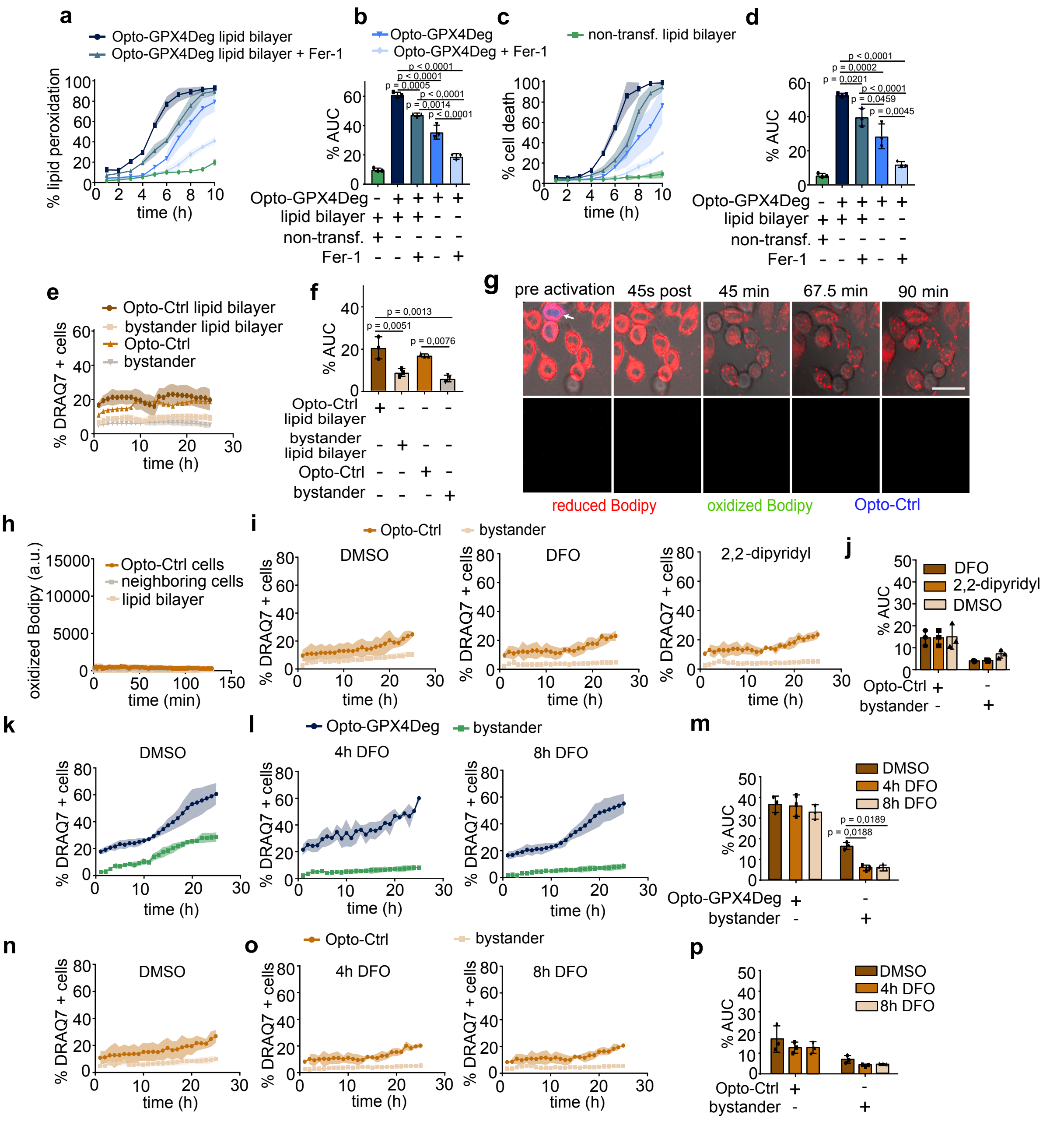

Supplementary Figure 11: Controls for experiments with cells seeded on a supported lipid bilayer and with DFO. (a-d) Kinetics of C11-Bodipy oxidation **(a)** and cell death **(c)** for activated Opto-GPX4Deg and bystander HeLa cells grown on glass or on a supported lipid bilayer and treated or not with 5 µM Fer-1. All experiments were performed with three independent biological replicates (n=3). Values are displayed as mean ± SD. **(b, d)** %AUC for the indicated cell populations. **(e)** Kinetics of cell death for activated Opto-Ctrl HeLa and bystander cells grown on glass or on a supported lipid bilayer and treated or not with 5 µM Fer-1. **(f)** %AUC for the indicated cell populations. All experiments were performed with three independent biological replicates (n=3). Values are displayed as mean ± SD. **(g)** Time series of C11-Bodipy oxidation and cell death in activated Opto-Ctrl HeLa cells and bystander neighboring cells grown on a lipid bilayer. Opto-Ctrl, blue; oxidized C11-Bodipy, green; reduced

C11-Bodipy, red. Scale bar, 30 µm. **(h)** Quantification of **(g)** All experiments were performed with three independent biological replicates (n=3). Values are displayed as mean ± SD. **(i)** Kinetics of cell death for activated Opto-Ctrl and bystander HeLa cells treated with DMSO, 100 µM DFO or 100 µM 2,2dipyridyl as indicated. **(j)** %AUC for the indicated cell populations. Potential endosomal uptake and accumulation of DFO does not interfere with the intrinsic ferroptosis sensitivity of cells. All experiments were performed with three independent biological replicates (n=3). Values are displayed as mean ± SD. **(k,l,n,o)** Kinetics of cell death for activated Opto-GPX4Deg or Opto-Ctrl HeLa cells pretreated for 4h or 8h hours with DMSO or 100 µM DFO. **(m,p)** %AUC for the indicated cell populations. In all experiments, statistical analysis by two-sided one-way ANOVA corrected for multiple comparisons using Tukey's multiple comparison test. Exact p-values are shown. All experiments were performed with three independent biological replicates (n=3). Values as mean ± SD.

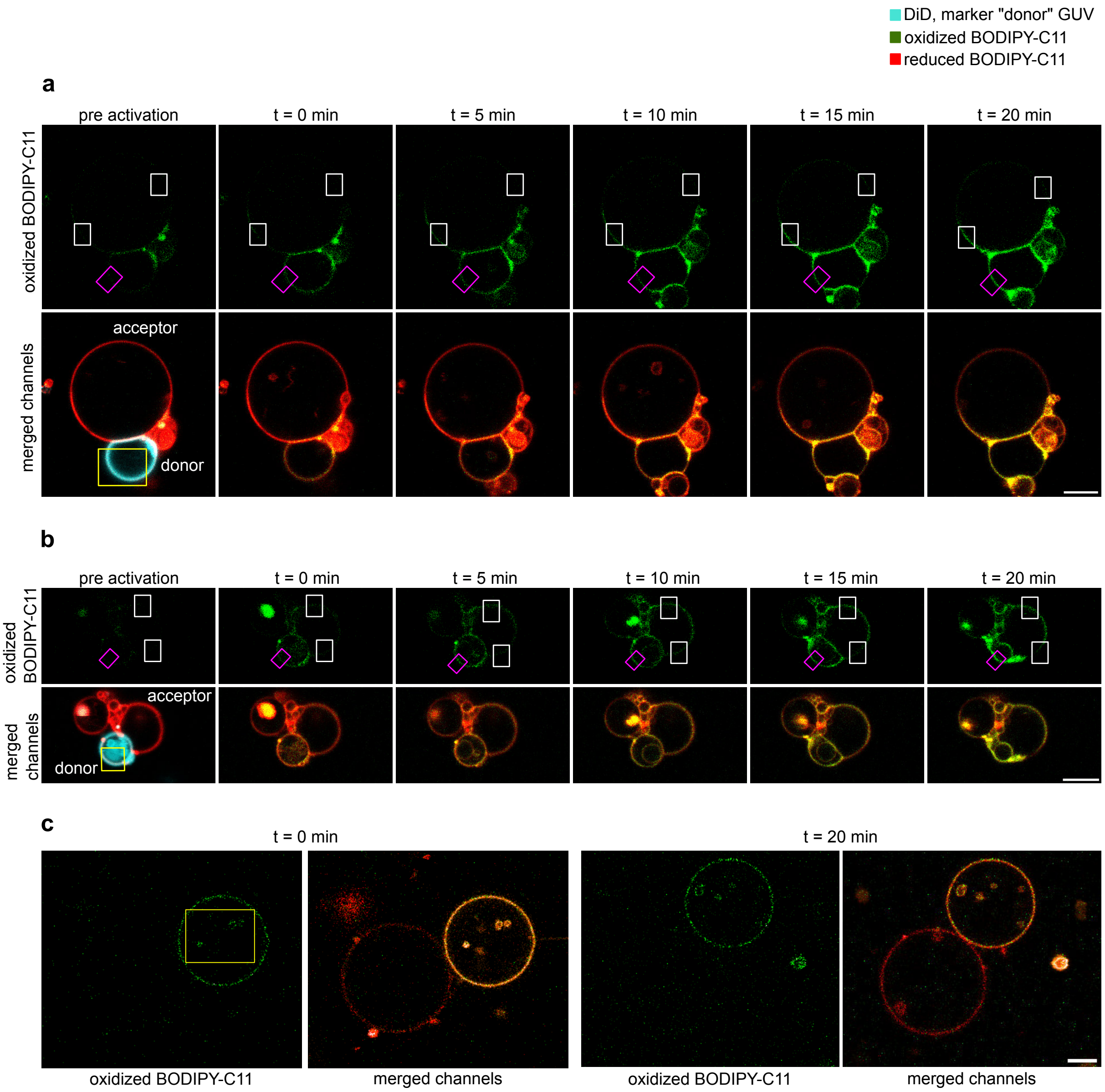

Supplementary Figure 12: Lipid peroxidation can propagate between contacting GUVs. (a and b) Representative time series of oxidation propagation between contacting GUVs induced by addition of biotinylated lipids in the membrane and streptavidin to the medium. Oxidized C11-Bodipy shown in green; reduced C11-Bodipy shown in red; DID and 1,9-dimethyl methylene blue (DMMB) are shown in blue and label donor GUVs. Controlled illumination in a small membrane region of the donor GUV (yellow box) induced light-activated lipid oxidation in the donor GUV and later on in the acceptor GUV. t = 0 min indicates first image after activation. Membrane regions in which the C11-Bodipy oxidation ratio was calculated are shown in white (acceptor GUV) and pink (donor GUV) boxes. All experiments were performed with three independent biological replicates (n=3). Scale bar, 5 µm. **(c)** Control experiment. We used GUVs lacking contacting membrane areas made of PC:PS:PAPC (90:5:5) and PC:PAPC:DOTAP:DMMB (90:5:2:5), as a strategy to get them close to each other via electrostatic interactions. Under these conditions, illumination of the donor GUVs containing DMMB caused lipid oxidation in that same GUV, but did not lead to propagation of lipid oxidation to the acceptor GUVs. The yellow box indicates the membrane region of the “donor” GUV that was light activated. t = 0 min indicates first

image after activation. All experiments were performed with three independent biological replicates (n=3). Scale bar, 5 μm.

a

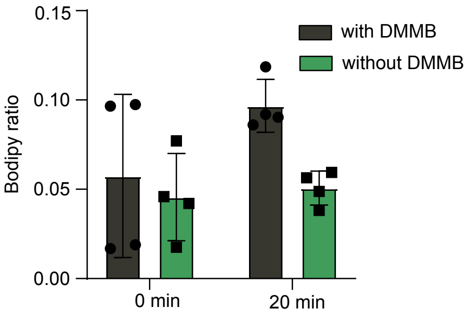

Supplementary Figure 13: GUV imaging without photoactivation did not result in lipid oxidation. (a) Quantification of the ratio between oxidized and reduced C11-Bodipy in GUVs over time in absence or presence of DMMB. Statistical analysis by two-sided two-way ANOVA corrected for multiple comparisons using Tukey's multiple comparison test. Exact p-values are shown. All experiments were performed with three independent biological replicates (n=4). Values are displayed as mean ± SD.
