# Supplementary material for: Ferroptosis spreads to neighboring cells via plasma membrane contacts
Source: Nat Commun. 2025 Mar 26;16:2951. doi: 10.1038/s41467-025-58175-w (PMC11947162; doi:10.1038/s41467-025-58175-w)
Supplement: Supplementary file 2 — Description of Addtional Supplementary Files [file 41467_2025_58175_MOESM2_ESM.pdf]

Description of Additional Supplementary Files

Supplementary movie 1: Cell death propagation upon light-driven ferroptosis induction. Live cell imaging assessing cell death (DRAQ7, magenta) in bystander HeLa cells (GFP negative) over 24h in Opto-GPX4Deg and Opto-Ctrl samples using an IncuCyte. Scale bar, 100 µm.

Supplementary movie 2: Lipid peroxidation and ferroptotic cell death propagates to neighboring cells. (a-c) Representative videos for C11-Bodipy oxidation (green) and cell death in activated Opto-Ctrl- (a), or in Opto-GPX4Deg samples treated or not with 5µM Fer-1 (b,c, respectively). Blue signal indicates Opto-GPX4Deg or Opto-Ctrl expression. (c) AI-derived alive/dead mask allows the assessment of cell death in microscopy images. Upper panel, blue signal indicates Opto-GPX4Deg expression, green signal indicates C11-Bodipy oxidation. Lower panel, cell death detection by AIbased software. Living cells are indicated in blue and dead cells in pink. Scale bar, 50 µm.

Supplementary movie 3: Lipid peroxidation and ferroptosis propagation is promoted by seeding cells on a lipid bilayer. Representative video of C11-Bodipy oxidation and cell death in photo-activated Opto-GPX4Deg HeLa cells and bystander neighboring cells seeded on a lipid bilayer. Opto-GPX4Deg, blue; oxidized Bodipy, green; reduced Bodipy, red. Scale bar, 30 µm.

Supplementary movie 4: Control experiment for ferroptosis propagation on a lipid bilayer. Representative video of C11-Bodipy oxidation and cell death in confocal activated Opto-Ctrl HeLa cells and bystander neighboring cells grown on a lipid bilayer. Opto-Ctrl, blue; oxidized Bodipy, green; reduced Bodipy, red. Scale bar, 20 µm.
