# Supplementary material for: Ferroptosis spreads to neighboring cells via plasma membrane contacts
Source: Nat Commun. 2025 Mar 26;16:2951. doi: 10.1038/s41467-025-58175-w (PMC11947162; doi:10.1038/s41467-025-58175-w)
Supplement: Supplementary file 7 — Reporting Summary [file 41467_2025_58175_MOESM7_ESM.pdf]

nature portfolio

Corresponding author(s): Ana J. Garcia-Saez

Last updated by author(s): Feb 18, 2025

Reporting Summary

Nature Portfolio wishes to improve the reproducibility of the work that we publish. This form provides structure for consistency and transparency in reporting. For further information on Nature Portfolio policies, see our Editorial Policies and the Editorial Policy Checklist.

Statistics

For all statistical analyses, confirm that the following items are present in the figure legend, table legend, main text, or Methods section.

n/a	Confirmed	
☐	☒	The exact sample size (*n*) for each experimental group/condition, given as a discrete number and unit of measurement
☐	☒	A statement on whether measurements were taken from distinct samples or whether the same sample was measured repeatedly
☐	☒	The statistical test(s) used AND whether they are one- or two-sided<br>*Only common tests should be described solely by name; describe more complex techniques in the Methods section.*
☒	☐	A description of all covariates tested
☐	☒	A description of any assumptions or corrections, such as tests of normality and adjustment for multiple comparisons
☐	☒	A full description of the statistical parameters including central tendency (e.g. means) or other basic estimates (e.g. regression coefficient) AND variation (e.g. standard deviation) or associated estimates of uncertainty (e.g. confidence intervals)
☐	☒	For null hypothesis testing, the test statistic (e.g. *F*, *t*, *r*) with confidence intervals, effect sizes, degrees of freedom and *P* value noted<br>*Give P values as exact values whenever suitable.*
☒	☐	For Bayesian analysis, information on the choice of priors and Markov chain Monte Carlo settings
☒	☐	For hierarchical and complex designs, identification of the appropriate level for tests and full reporting of outcomes
☒	☐	Estimates of effect sizes (e.g. Cohen's *d*, Pearson's *r*), indicating how they were calculated

Our web collection on statistics for biologists contains articles on many of the points above.

Software and code

Policy information about availability of computer code

Data collection	Lipidomics data were aquired using Analyst 1.7.3. software (SCIEX).
Data analysis	Lipidomics data were analyzed using LipidView 1.2 and MultiQuant 3.0.3 softwares (SCIEX). Incucyte experiments were either analyzed by Incucyte Cell-by-Cell Analysis Software Module (Sartorius) or a custom made software. Confocal microscopy experiments were analyzed using ImageJ/FIJI (https://doi.org/10.1038/nmeth.2019). All statistical tests were performed using GraphPad Prism 7 (GraphPad Software).

For manuscripts utilizing custom algorithms or software that are central to the research but not yet described in published literature, software must be made available to editors and reviewers. We strongly encourage code deposition in a community repository (e.g. GitHub). See the Nature Portfolio guidelines for submitting code & software for further information.

Data

Policy information about availability of data

All manuscripts must include a data availability statement. This statement should provide the following information, where applicable:

- Accession codes, unique identifiers, or web links for publicly available datasets
- A description of any restrictions on data availability
- For clinical datasets or third party data, please ensure that the statement adheres to our policy

Source data are provided with this paper. Lipidomics data have been deposited in the Zenodo data repository and can be downloaded via https://doi.org/10.5281/zenodo.14905646. All other data supporting the findings of this manuscript are available from the corresponding author upon request. Access to the data will be

granted upon request for academic or non-commercial research purposes, provided that the requester agrees to maintain confidentiality (if applicable), properly cite the source, comply with any legal or ethical restrictions, and that fulfilling the request does not impose an undue burden on the authors.

Research involving human participants, their data, or biological material

Policy information about studies with human participants or human data. See also policy information about sex, gender (identity/presentation), and sexual orientation and race, ethnicity and racism.

Reporting on sex and gender	*Use the terms sex (biological attribute) and gender (shaped by social and cultural circumstances) carefully in order to avoid confusing both terms. Indicate if findings apply to only one sex or gender; describe whether sex and gender were considered in study design; whether sex and/or gender was determined based on self-reporting or assigned and methods used. Provide in the source data disaggregated sex and gender data, where this information has been collected, and if consent has been obtained for sharing of individual-level data; provide overall numbers in this Reporting Summary. Please state if this information has not been collected. Report sex- and gender-based analyses where performed, justify reasons for lack of sex- and gender-based analysis.*
Reporting on race, ethnicity, or other socially relevant groupings	*Please specify the socially constructed or socially relevant categorization variable(s) used in your manuscript and explain why they were used. Please note that such variables should not be used as proxies for other socially constructed/relevant variables (for example, race or ethnicity should not be used as a proxy for socioeconomic status). Provide clear definitions of the relevant terms used, how they were provided (by the participants/respondents, the researchers, or third parties), and the method(s) used to classify people into the different categories (e.g. self-report, census or administrative data, social media data, etc.) Please provide details about how you controlled for confounding variables in your analyses.*
Population characteristics	*Describe the covariate-relevant population characteristics of the human research participants (e.g. age, genotypic information, past and current diagnosis and treatment categories). If you filled out the behavioural & social sciences study design questions and have nothing to add here, write "See above."*
Recruitment	*Describe how participants were recruited. Outline any potential self-selection bias or other biases that may be present and how these are likely to impact results.*
Ethics oversight	*Identify the organization(s) that approved the study protocol.*

Note that full information on the approval of the study protocol must also be provided in the manuscript.

Field-specific reporting

Please select the one below that is the best fit for your research. If you are not sure, read the appropriate sections before making your selection.

☒ Life sciences ☐ Behavioural & social sciences ☐ Ecological, evolutionary & environmental sciences

For a reference copy of the document with all sections, see nature.com/documents/nr-reporting-summary-flat.pdf

Life sciences study design

All studies must disclose on these points even when the disclosure is negative.

Sample size	*Describe how sample size was determined, detailing any statistical methods used to predetermine sample size OR if no sample-size calculation was performed, describe how sample sizes were chosen and provide a rationale for why these sample sizes are sufficient.*
Data exclusions	One replicate of the lipidomics analysis was excluded, because sample preparation of this replicate did not sufficiently work according to WB analysis.
Replication	All experiments were performed measuring at least duplicates during each individual experiment and means were calculated. These experiments were repeated independently at least three times to obtain at least three indepent means. All figures show means calculated from these three means +/- SD. All experimental replicates sucessfully validated the experimental findings.
Randomization	*Describe how samples/organisms/participants were allocated into experimental groups. If allocation was not random, describe how covariates were controlled OR if this is not relevant to your study, explain why.*
Blinding	*Describe whether the investigators were blinded to group allocation during data collection and/or analysis. If blinding was not possible, describe why OR explain why blinding was not relevant to your study.*

Reporting for specific materials, systems and methods

We require information from authors about some types of materials, experimental systems and methods used in many studies. Here, indicate whether each material, system or method listed is relevant to your study. If you are not sure if a list item applies to your research, read the appropriate section before selecting a response.

Materials & experimental systems

n/a	Involved in the study
	☒ Antibodies
	☒ Eukaryotic cell lines
☒	Palaeontology and archaeology
☒	Animals and other organisms
☒	Clinical data
☒	Dual use research of concern
☒	Plants

Methods

n/a	Involved in the study
☒	ChIP-seq
☒	Flow cytometry
☒	MRI-based neuroimaging

Antibodies

Antibodies used	Anti-GPX4 (Abcam, # ab125066, clone EPNCIR144, Lot-GR3369574-7),<br>Anti-GFP antibody (Abcam, # ab290, polyclonal),<br>Anti-α-catenin (Sigma-Aldrich, # C2081, polyclonal, Lot-0000145845),<br>Anti-α-E-Catenin (D9R5E) (Cell Signaling, #36611, Lot-2)<br>Anti-FSP1 (PTG Lab, # 20886-1-AP, polyclonal, Lot-00101489),<br>Anti-YAP (Cell Signaling, #14074, clone D8H1X, Lot-5),<br>Anti-phospho-YAP (Ser127) (Cell Signaling, #13008, clone D9W2I, Lot-6),<br>Anti-GAPDH (Santa Cruz Biotechnology, sc-47724, clone 0411, Lot-12420),<br>Anti-VDAC2 (PTG Lab, 11663-1-AP, polyclonal),<br>Anti-ß-Actin (Santa Cruz Biotechnology, sc-47778, clone C4, Lot-E0720),<br>Anti-ß-tubulin (Santa Cruz Biotechnology, # sc-55529, clone G8, Lot-F2812),<br>IRDye 800CW Donkey anti-Mouse IgG Secondary Antibody (LI-COR, # 926-32212),<br>IRDye 680RD Donkey-anti-Rabbit Antibody (LI-COR, # 926-68023),<br>Goat anti-Rabbit IgG (H+L) Cross-Adsorbed Secondary Antibody, Alexa Fluor™ 488 (Thermo Fisher Scientific, A-11008, polyclonal, Lot-2420730)
Validation	Validation (for species; applications)<br>Anti-GPX4 (H/M/R; WB, IHC, IF), has been validated by supplier.<br>Anti-GFP (species independent; ELISA, IHC, IP, WB, Electron Microscopy), has been validated by supplier.<br>Anti-α-catenin (H/M/R; WB, IP, IF IHC) has been validated by supplier.<br>Anti-α-E-Catenin (H/M/R; WB, IF) has been validated by supplier.<br>Anti-FSP1 (H/M/R; WB, IP, IHC, IF, ELISA), has been validated by supplier.<br>Anti-YAP (H/M/R; WB, IP, IHC, IF, F, ChIP), has been validated by supplier.<br>Anti-phospho-YAP (H/M/R; WB, IP, IHC), has been validated by supplier.<br>Anti-GAPDH (H/M/R; WB, IHC), has been validated by supplier.<br>Anti-VDAC2 (H/M/R; WB, IP, IHC, IF, ELISA), has been validated by supplier.<br>Anti-ß-Actin (H/M/R/A; WB, IP, IF, IHC, ELISA), has been validated by supplier.<br>Anti-ß-tubulin (H/M/R/A; WB, IP, IF, IHC, ELISA), has been validated by supplier.<br>*Abbreviations: H-Human, M-Mouse, R-Rat, A-Avian, WB-Western Blot, IP-Immunoprecipitation, IHC-Immunohistochemestry, IF-Immunofluorescence, F-Flow Cytometry

Eukaryotic cell lines

Policy information about cell lines and Sex and Gender in Research

Cell line source(s)	Human Embryonic Kidney Cells: HEK293 and (gift from F. Essmann, Univ. of Tübingen).<br>Human colon cancer cells: HT-29 cells (gift from F. Essmann, Univ. of Tübingen).<br>Human Cervical Adenocarcinoma cells: HeLa (gift from A. Villunger, Med. Univ. of Innsbruck)<br>Mouse embryonic fibroblast: TAM-inducible Gpx4−/− cells (Pfa1) (gift from M. Conrad, Helmolz Center Munich)<br>Murine small cell lung cancer cells: RP285.5 GPX4 KO SCLC cells (gift from S. von Karstedt, Univ. of Cologne)<br>Murine colorectal caner cells: CT26 (gift from M. Pasparakis, Univ. of Cologne)<br>Murine melanoma cells: B16-F10 (gift from M. Pasparakis, Univ. of Cologne)
Authentication	None of the cell lines used were authenticated
Mycoplasma contamination	All cell lines in this study were subjected to regular mycoplasma testing using a kit (MycoStrip; Invivogen)
Commonly misidentified lines<br>(See ICLAC register)	None of the cell lines used are commonly misidentified lines.
