# Supplementary figures and images for: Ferroptosis spreads to neighboring cells via plasma membrane contacts
Source: Nat Commun. 2025 Mar 26;16:2951. doi: 10.1038/s41467-025-58175-w (PMC11947162; doi:10.1038/s41467-025-58175-w)

Fig. S1L

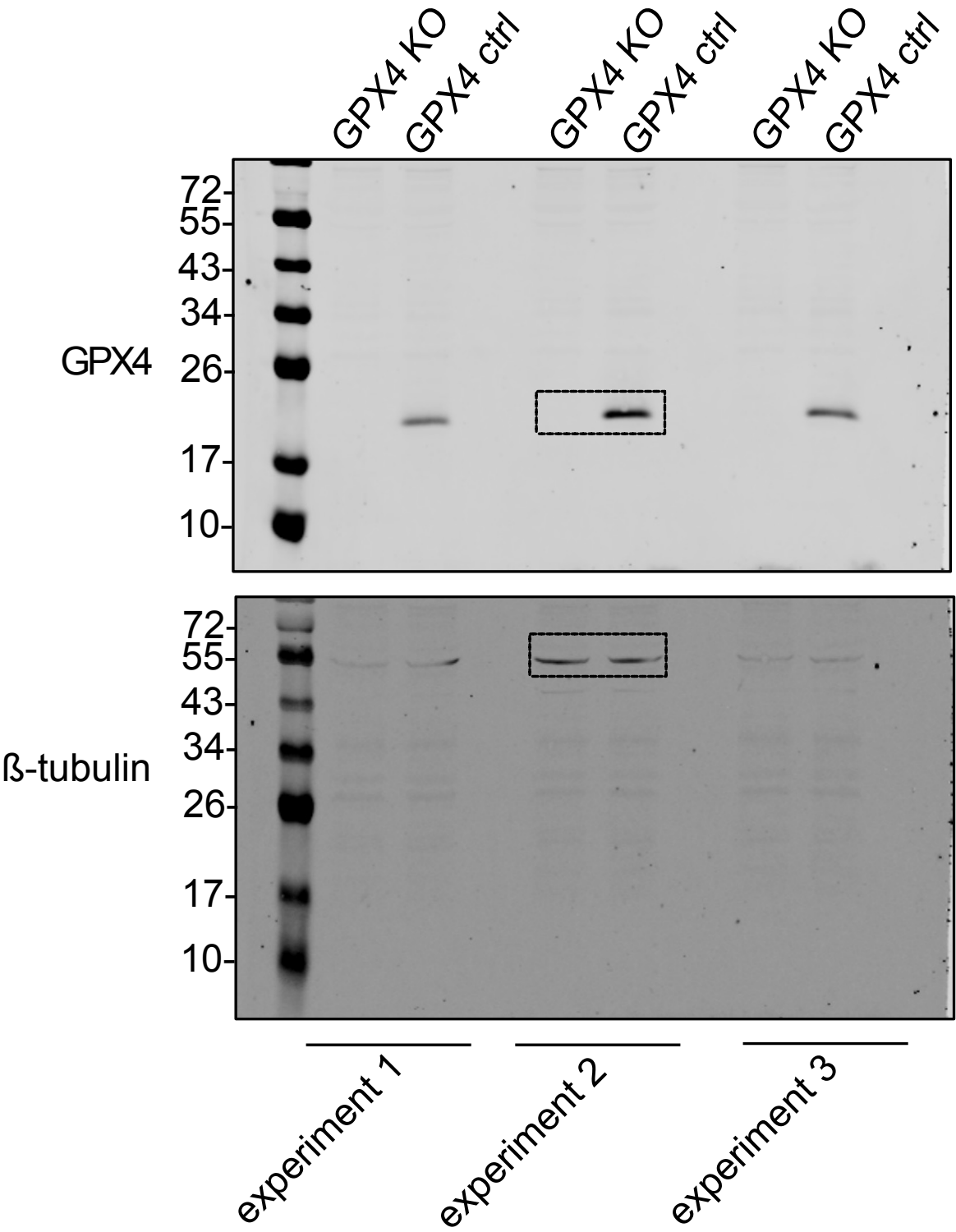

Fig. S2B

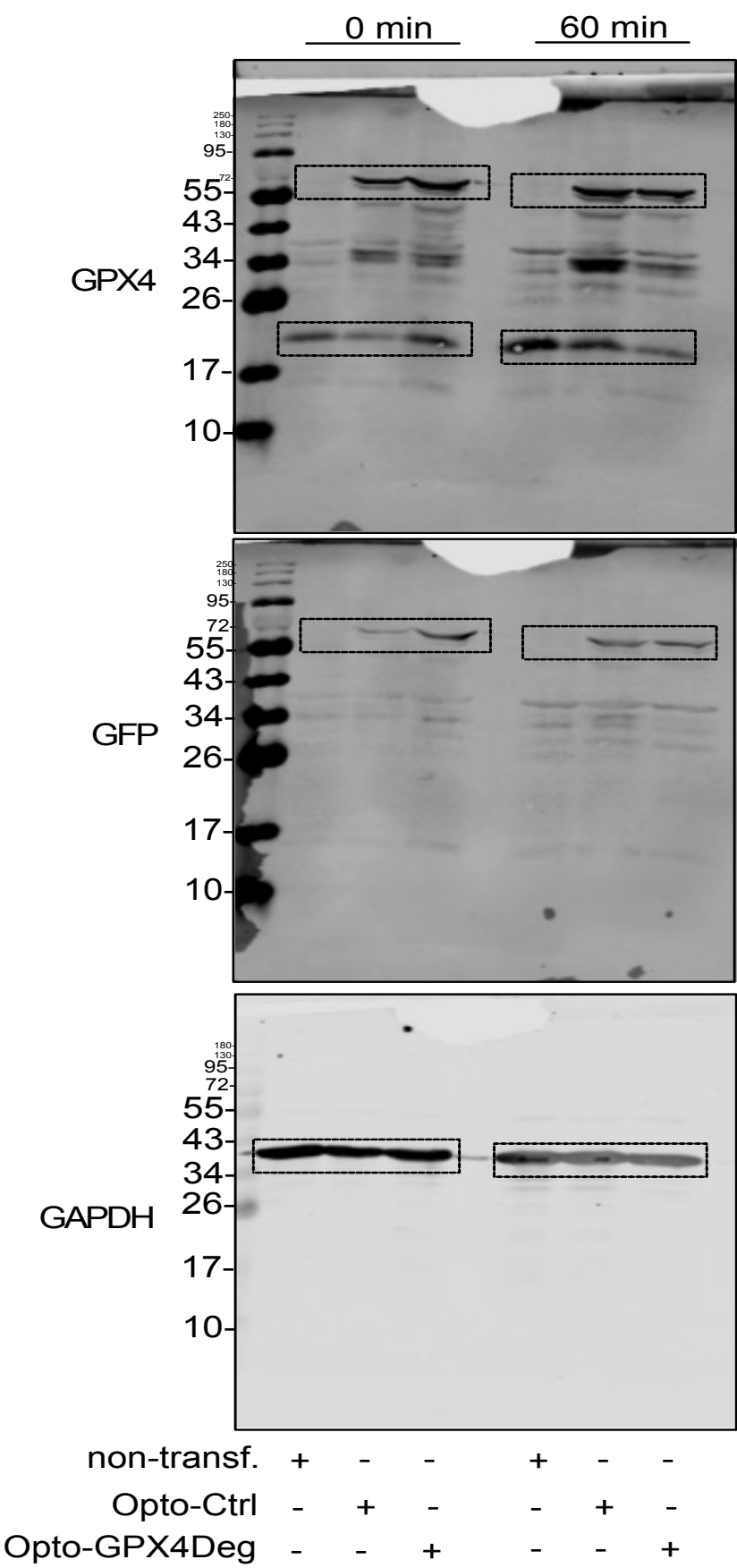

Fig. S6C

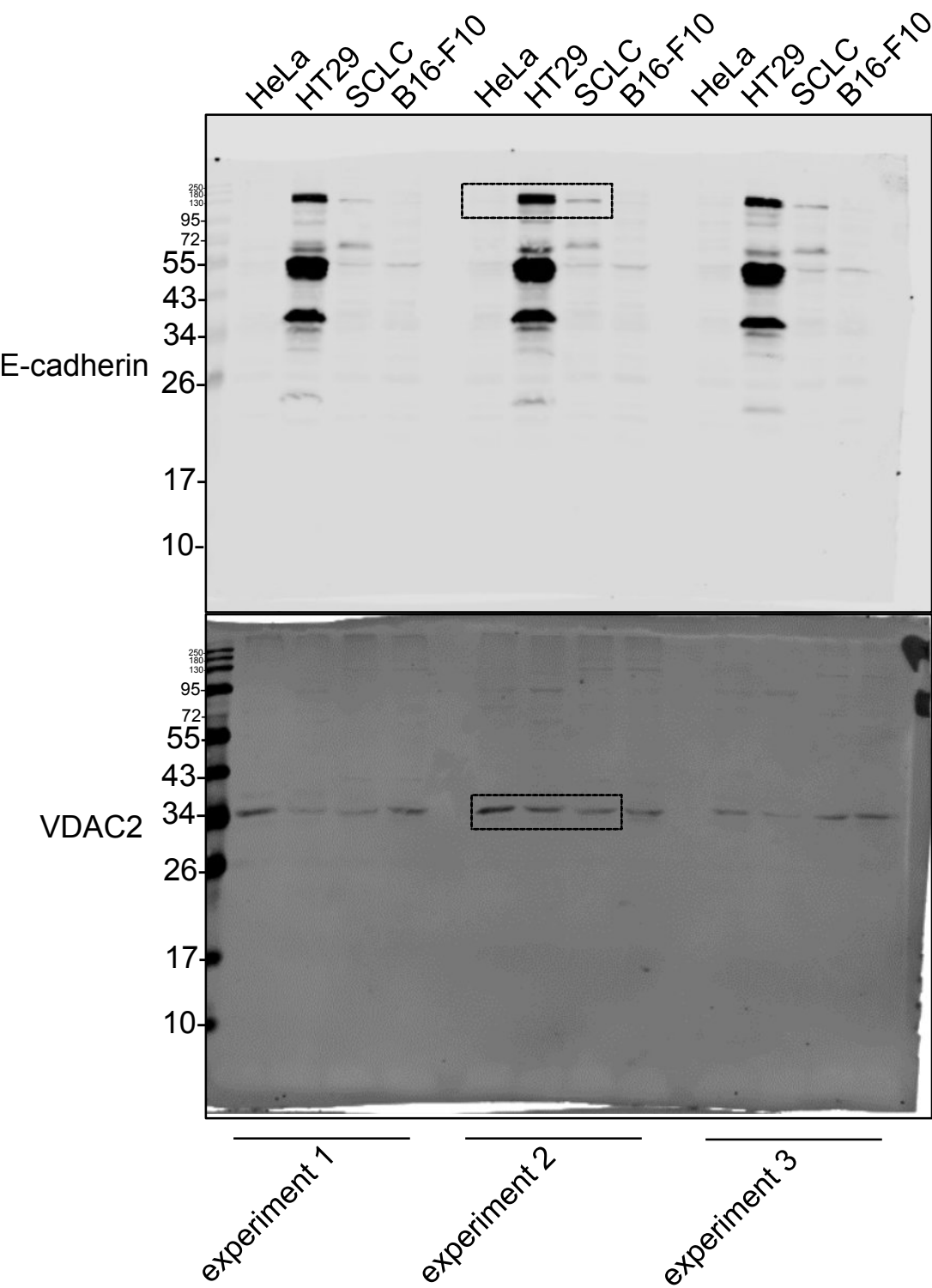

Fig. S8M

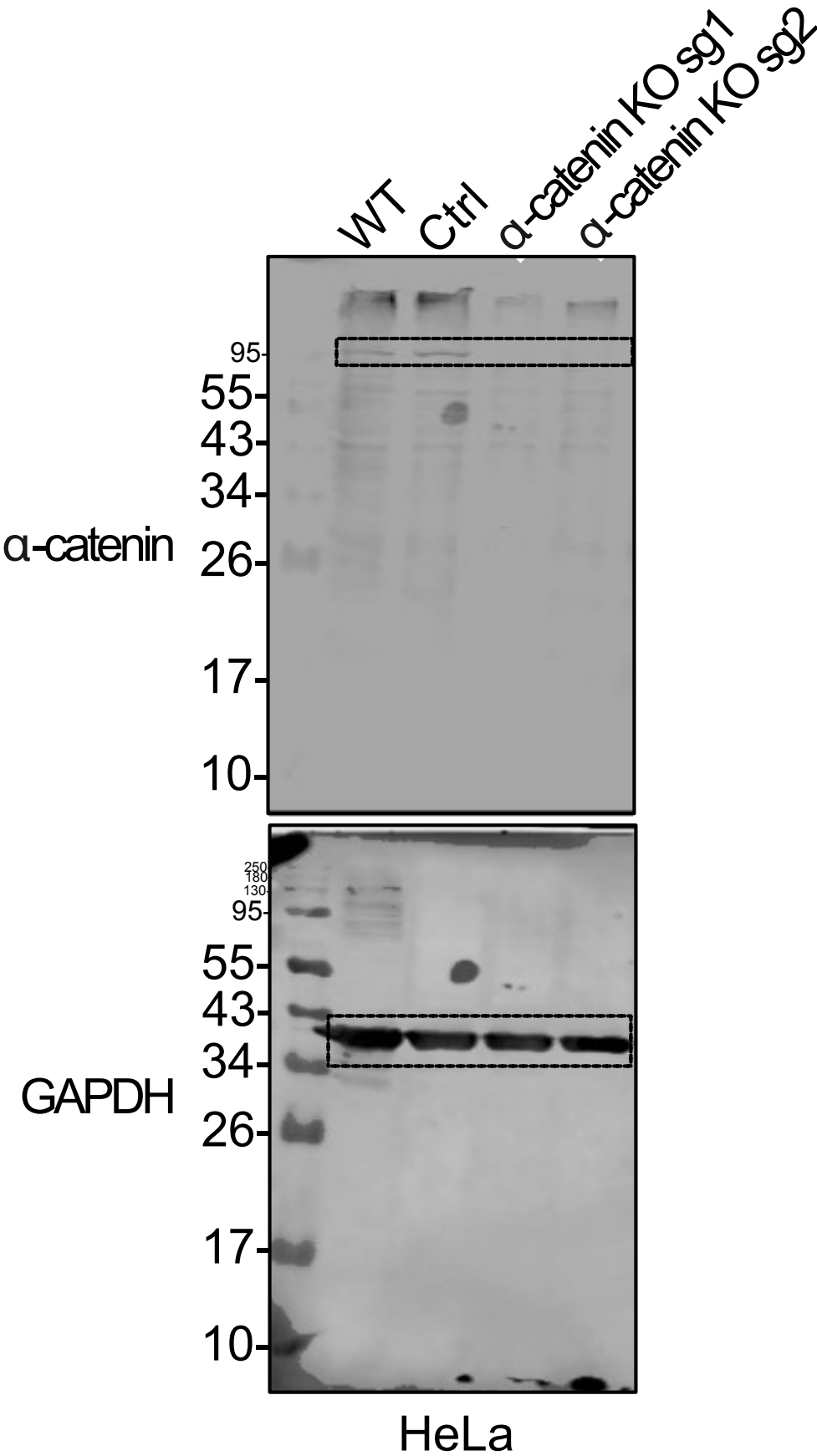

Fig. S8N

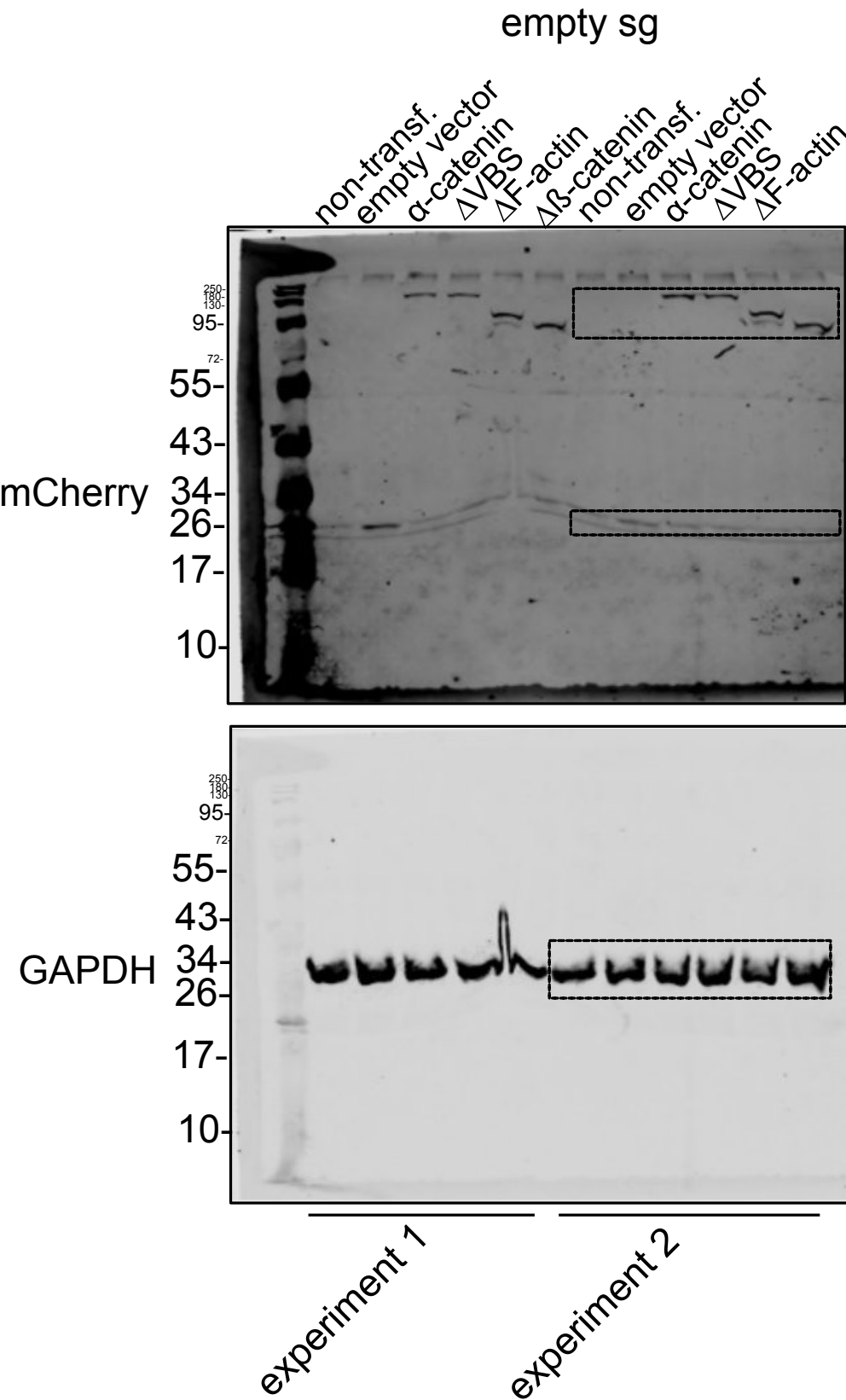

Fig. S8O

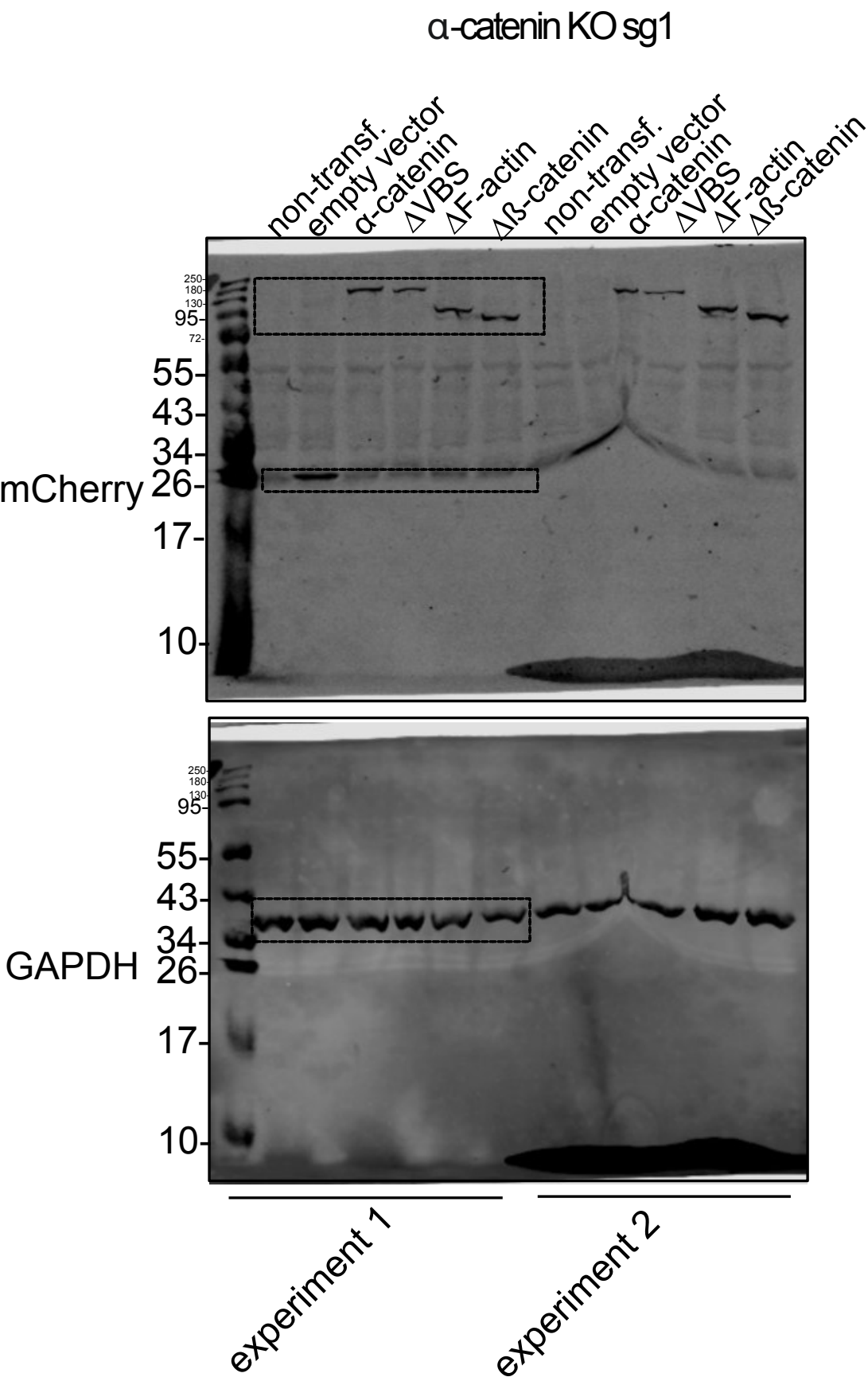

Fig. S8P

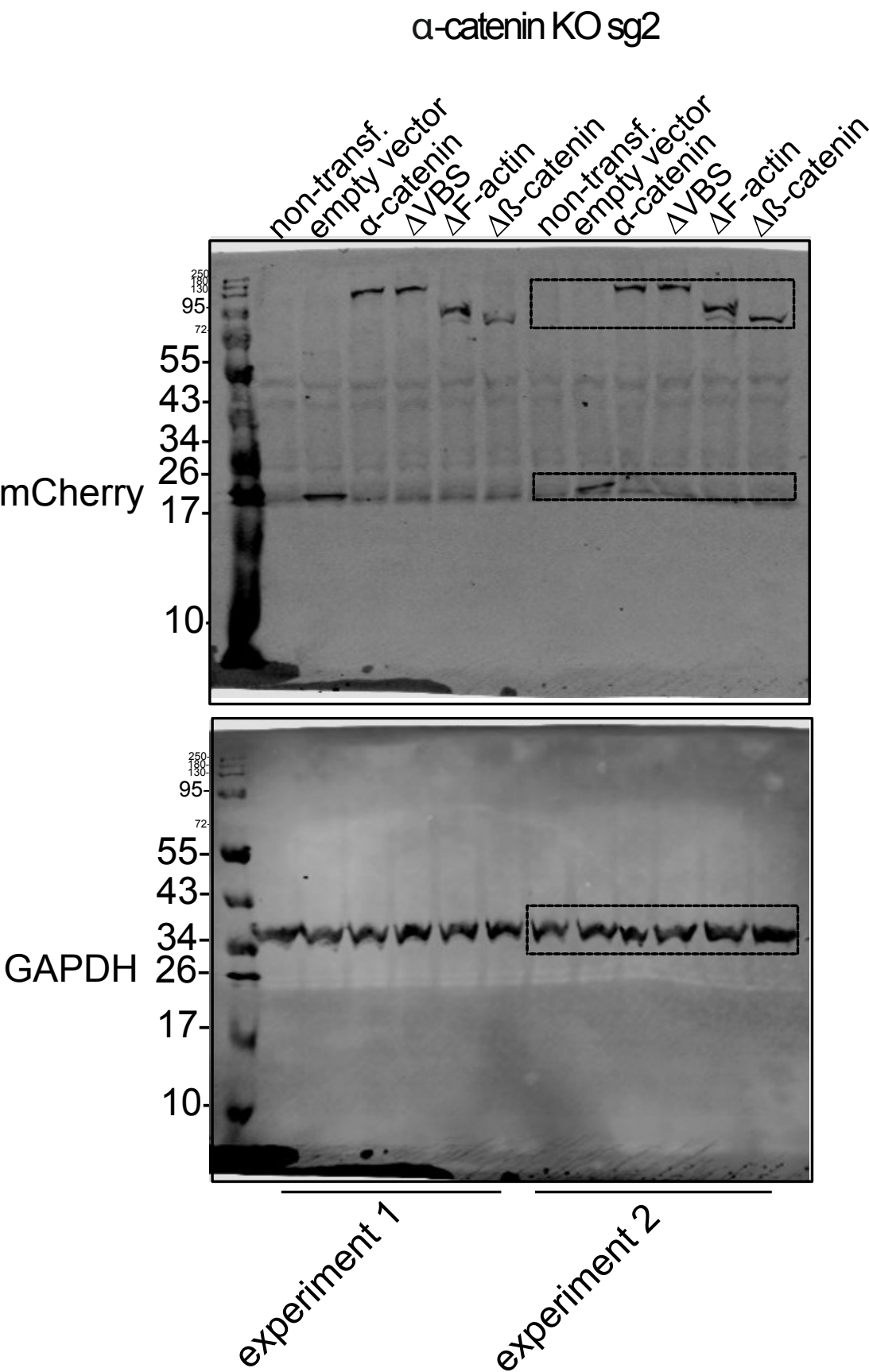

Fig. S10B

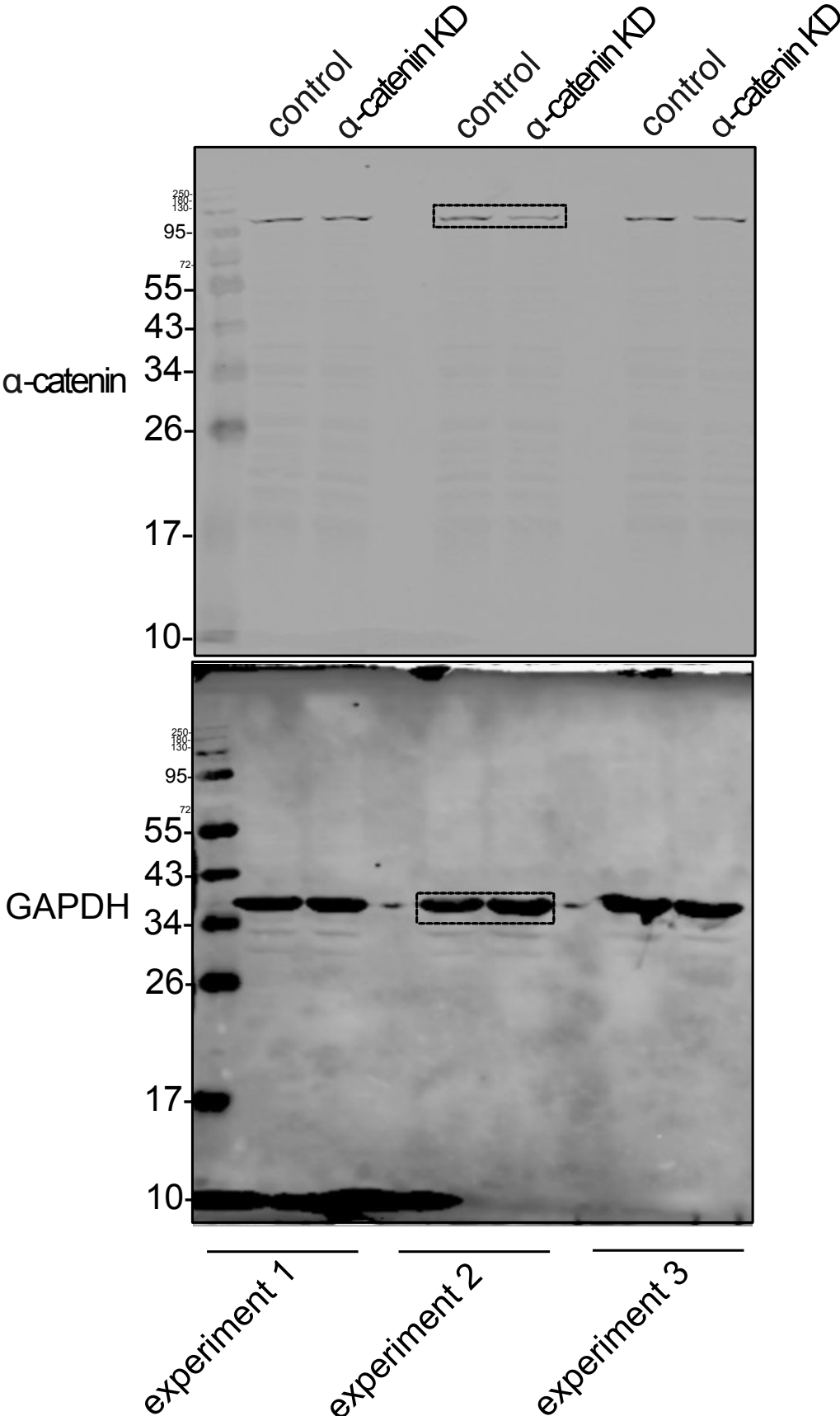

Fig. 1C

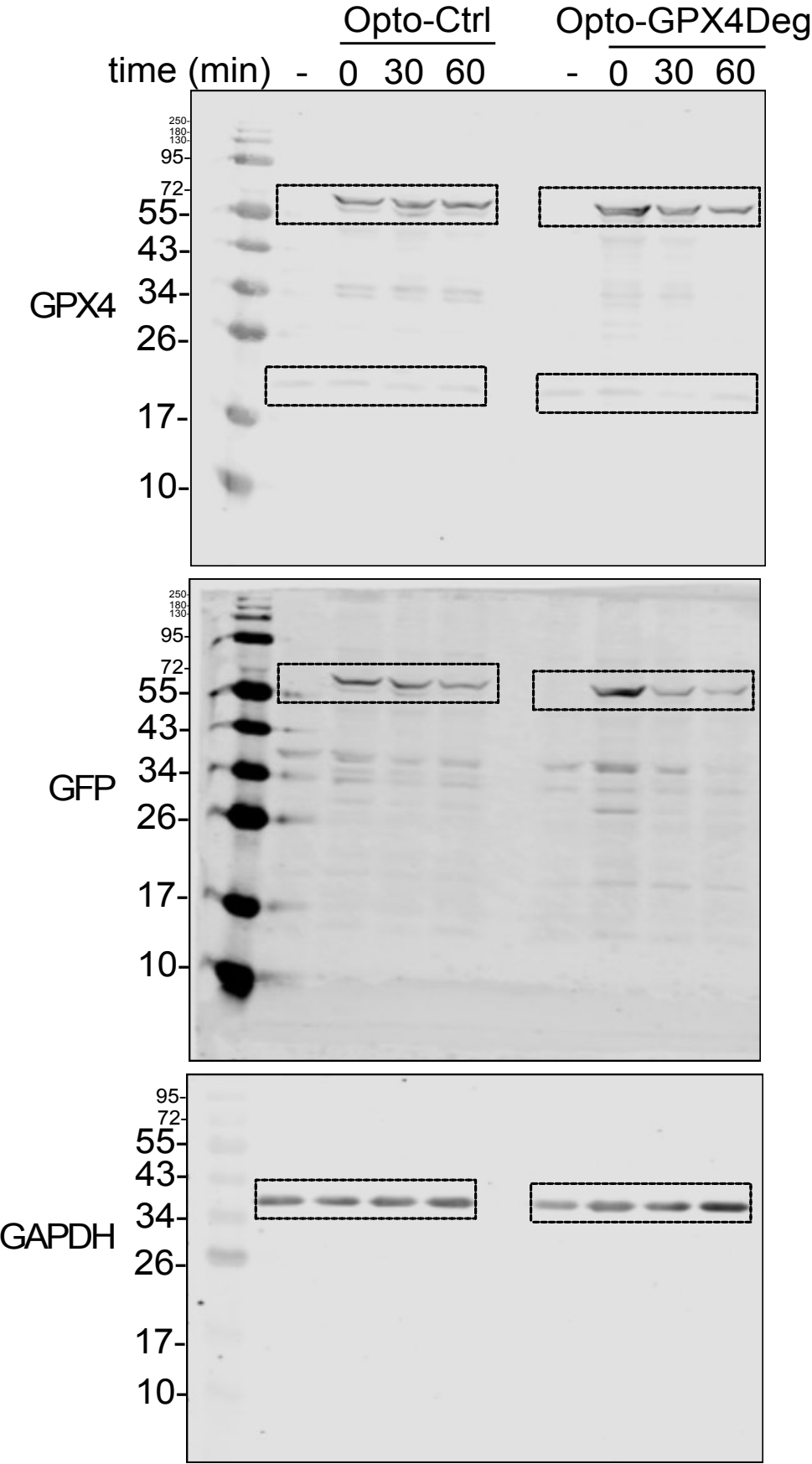

Fig. 5C

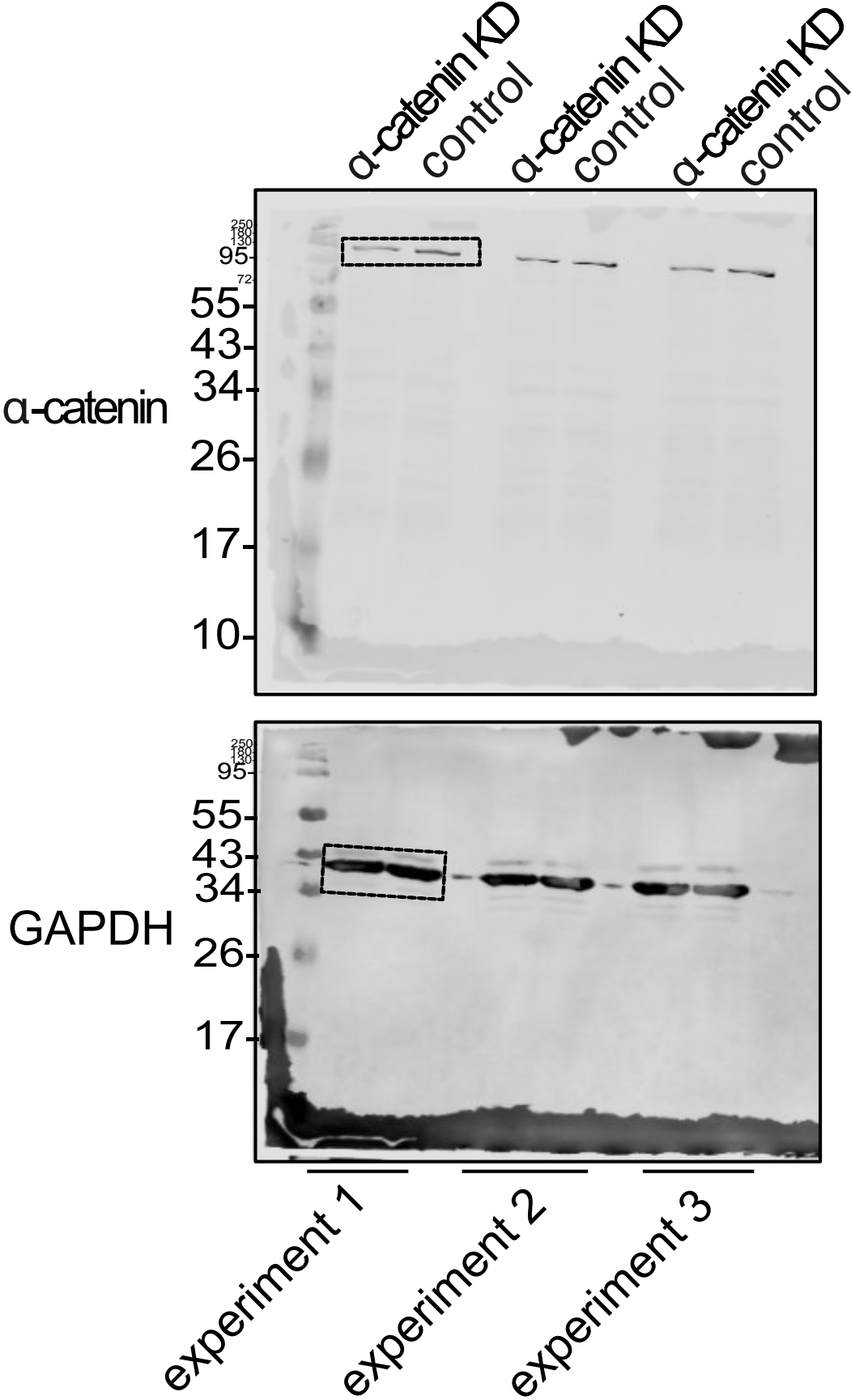

Fig.5C

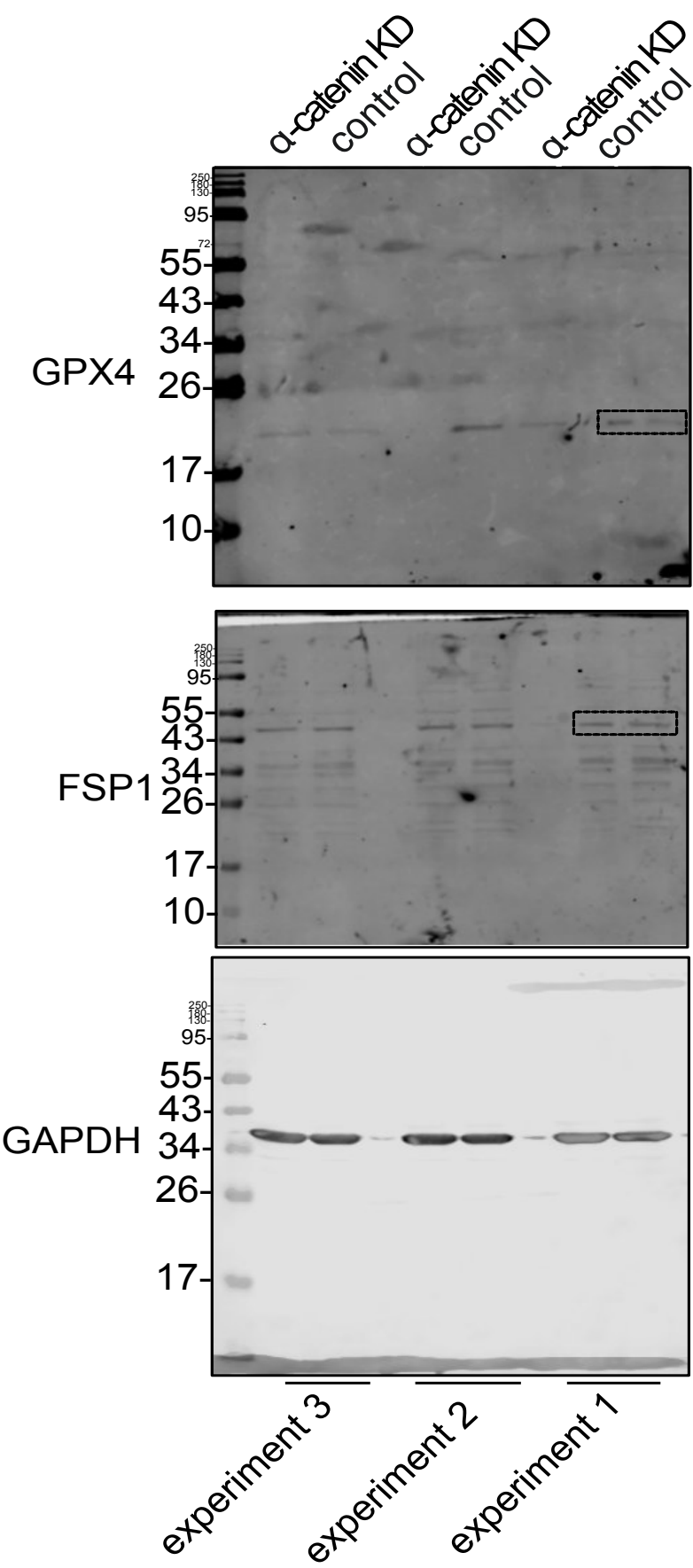

Fig. 5C

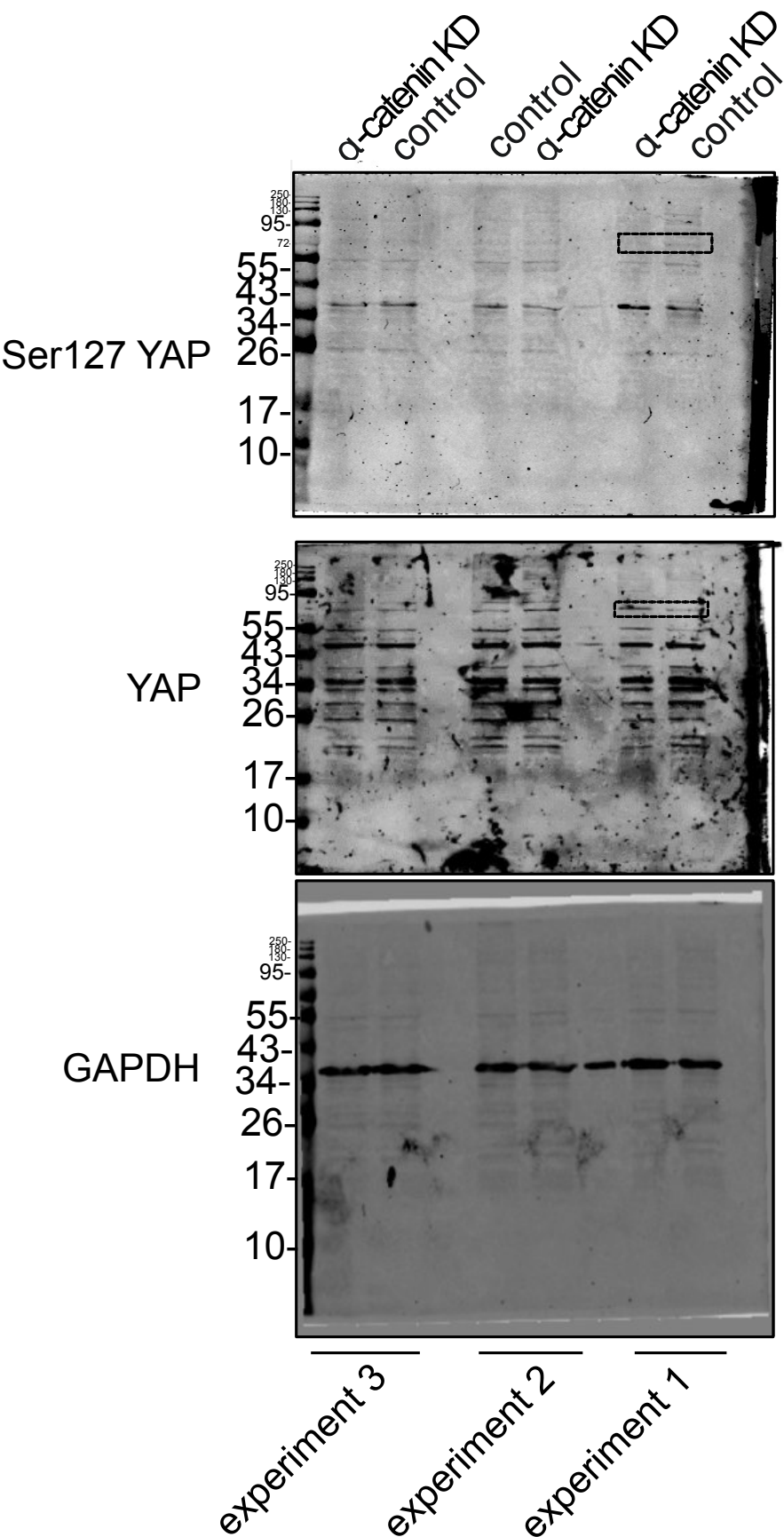

Fig. 5G

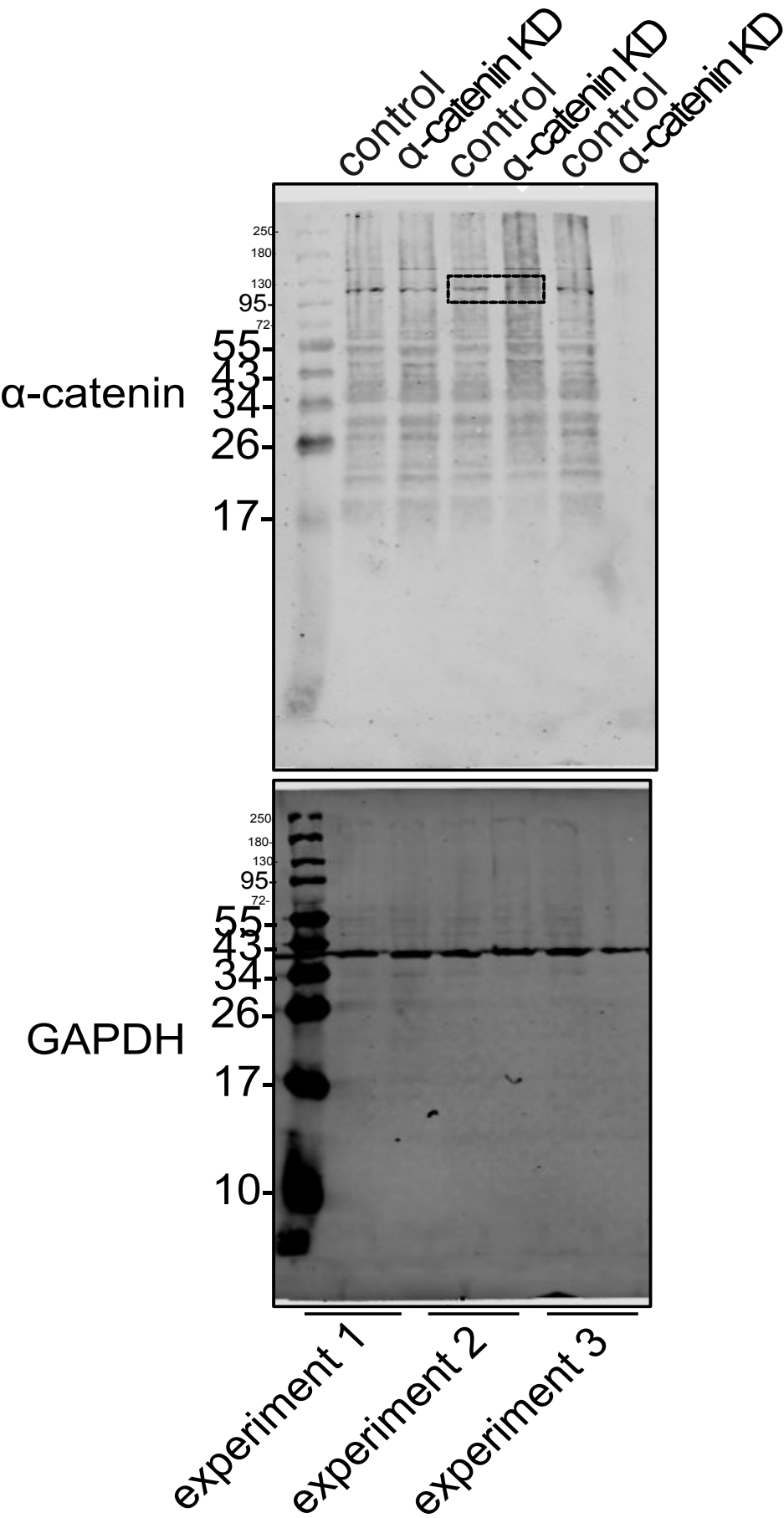

Fig. 5G

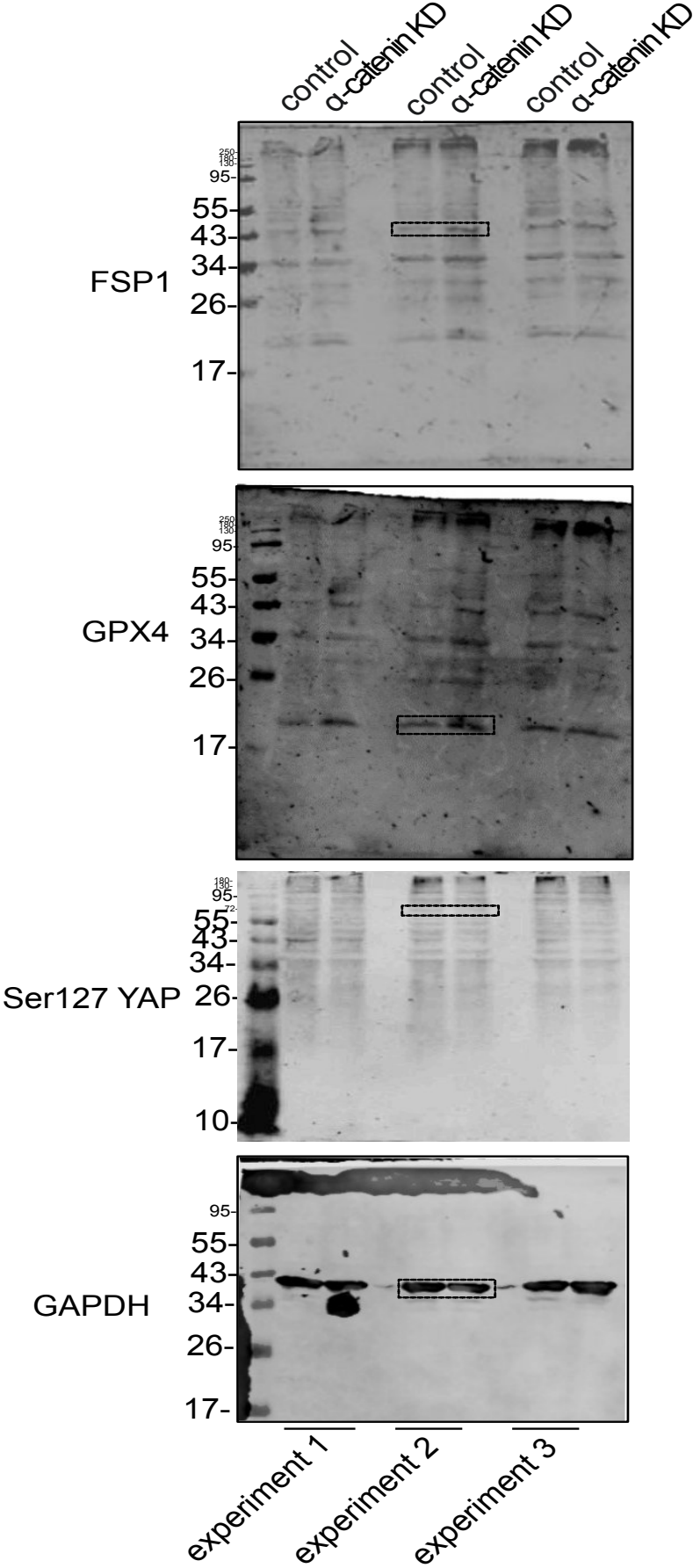

Fig. 5G

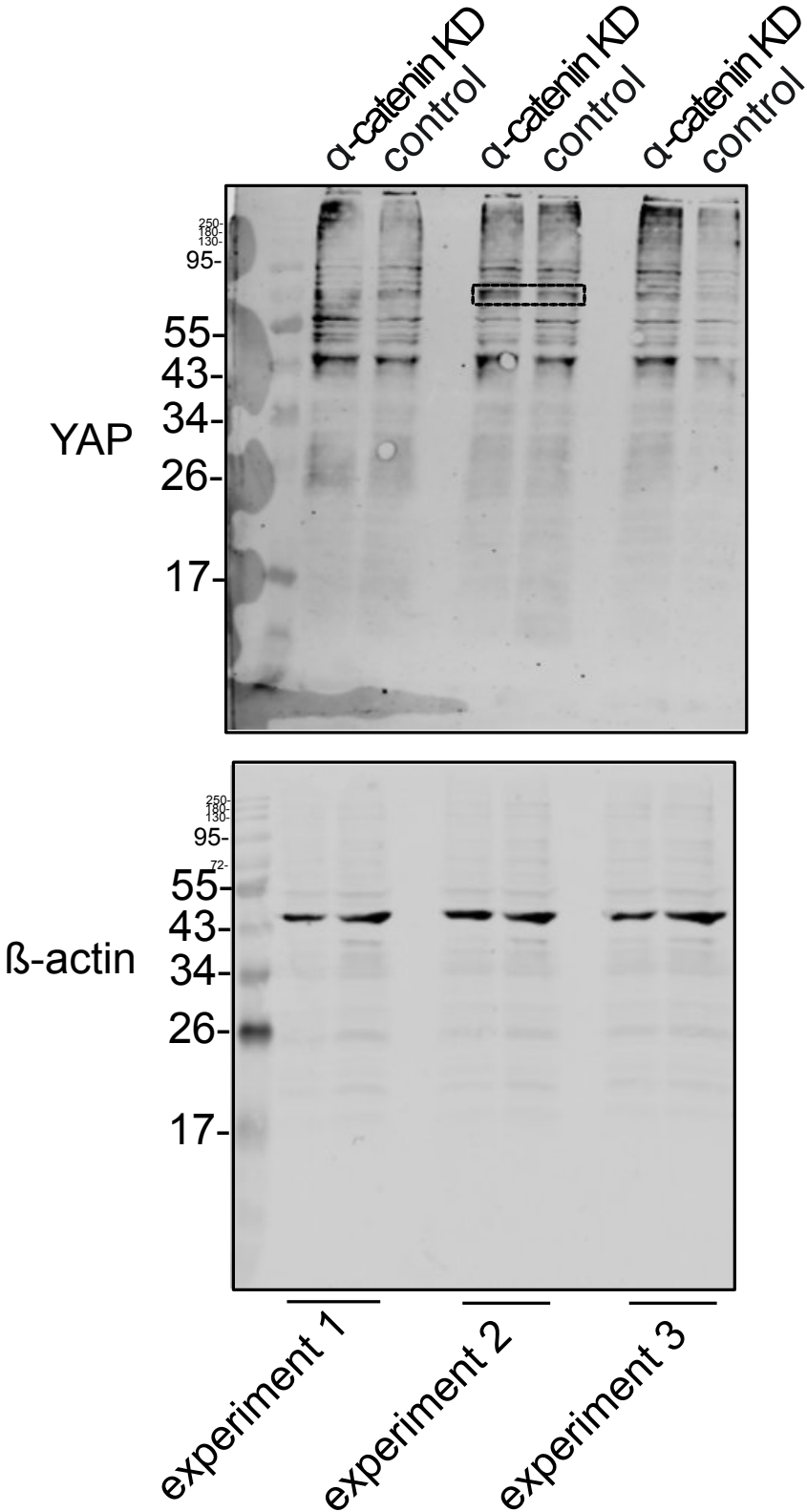

Fig. 5K

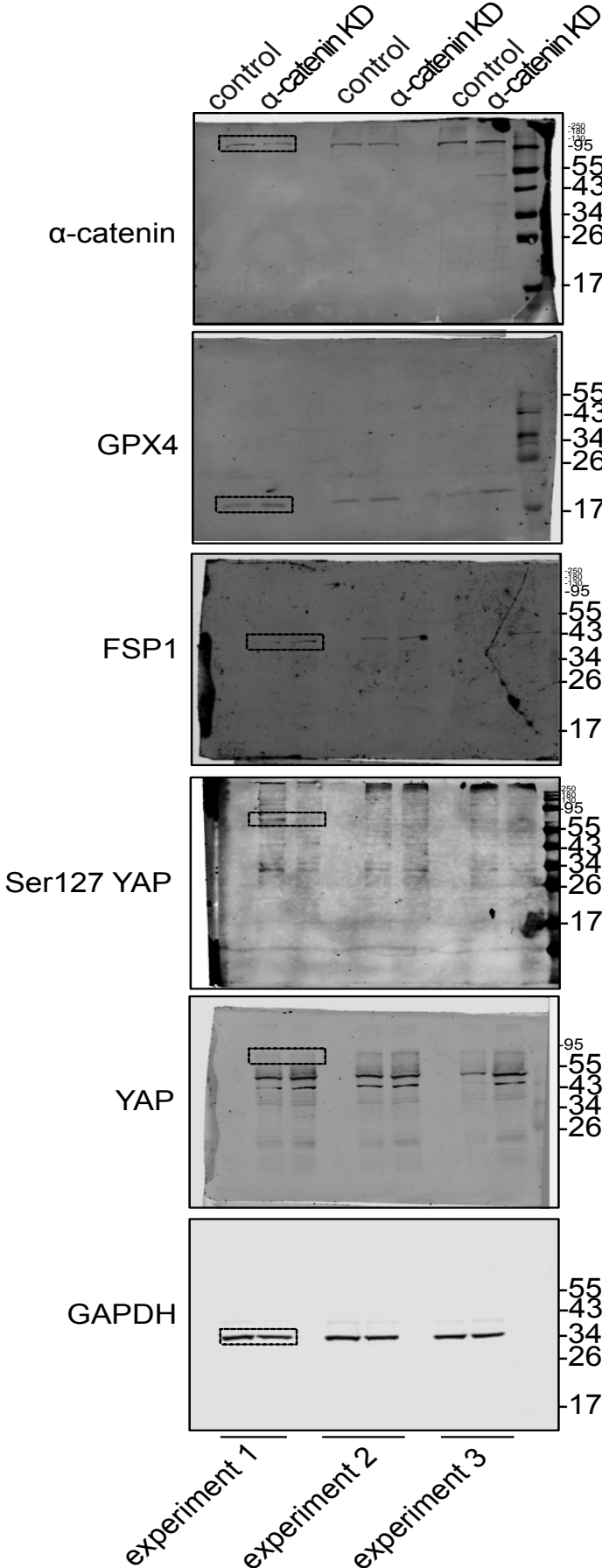

Supplement: Supplementary file 9 — Source Data [file 41467_2025_58175_MOESM9_ESM.zip › uncropped WB final submission.pdf]
